# MAST: a flexible statistical framework for assessing transcriptional changes and characterizing heterogeneity in single-cell RNA sequencing data

**DOI:** 10.1186/s13059-015-0844-5

**Published:** 2015-12-10

**Authors:** Greg Finak, Andrew McDavid, Masanao Yajima, Jingyuan Deng, Vivian Gersuk, Alex K. Shalek, Chloe K. Slichter, Hannah W. Miller, M. Juliana McElrath, Martin Prlic, Peter S. Linsley, Raphael Gottardo

**Affiliations:** Vaccine and Infectious Disease Division, Fred Hutchinson Cancer Research Center, Seattle, WA 98109 USA; Benaroya Research Institute at Virginia Mason, Seattle, WA 98101 USA; Institute for Medical Engineering & Science, MIT, Boston, MA 01239-4307 USA; Department of Chemistry, MIT, Boston, MA 01239-4307 USA; Ragon Institute of MGH, MIT, & Harvard, Boston, MA 02139-3583 USA; Broad Institute of MIT & Harvard, Boston, MA 01242 USA; Public Health Sciences Division, Fred Hutchinson Cancer Research Center, Seattle, WA 98109 USA

**Keywords:** Bimodality, Cellular detection rate, Co-expression, Empirical Bayes, Generalized linear model, Gene set enrichment analysis

## Abstract

Single-cell transcriptomics reveals gene expression heterogeneity but suffers from stochastic dropout and characteristic bimodal expression distributions in which expression is either strongly non-zero or non-detectable. We propose a two-part, generalized linear model for such bimodal data that parameterizes both of these features. We argue that the cellular detection rate, the fraction of genes expressed in a cell, should be adjusted for as a source of nuisance variation. Our model provides gene set enrichment analysis tailored to single-cell data. It provides insights into how networks of co-expressed genes evolve across an experimental treatment. MAST is available at https://github.com/RGLab/MAST.

**Electronic supplementary material:**

The online version of this article (doi:10.1186/s13059-015-0844-5) contains supplementary material, which is available to authorized users.

## Background

Whole transcriptome expression profiling of single cells via RNA sequencing (scRNA-seq) is the logical apex to single cell gene expression experiments. In contrast to transcriptomic experiments on mRNA derived from bulk samples, this technology provides powerful multi-parametric measurements of gene co-expression at the single-cell level. However, the development of equally potent analytic tools has trailed the rapid advances in biochemistry and molecular biology, and several challenges need to be addressed to fully leverage the information in single-cell expression profiles.

First, single-cell expression has repeatedly been shown to exhibit a characteristic bimodal expression pattern, wherein the expression of otherwise abundant genes is either strongly positive or undetected within individual cells. This is due in part to low starting quantities of RNA such that many genes will be below the threshold of detection, but there is also a biological component to this variation (termed extrinsic noise in the literature) that is conflated with the technical variability [1–3]. We and other groups [4–7] have shown that the proportion of cells with detectable expression reflects both technical factors and biological differences between samples. Results from synthetic biology also support the notion that bimodality can arise from the stochastic nature of gene expression [2, 3, 8, 9].

Second, measuring single cell gene expression might seem to obviate the need to normalize for starting RNA quantities, but recent work shows that cells scale transcript copy number with cell volume (a factor that affects gene expression globally) to maintain a constant mRNA concentration and thus constant biochemical reaction rates [10, 11]. In scRNA-seq, cells of varying volume, and hence mRNA copy number, are diluted to an approximately fixed reaction volume, leading to differences in detection rates of various mRNA species that are driven by the initial cell volumes. Technical assay variability (e.g., mRNA quality, pre-amplification efficiency) and extrinsic biological factors (e.g., nuisance biological variability due to cell size) that globally affect transcription remain, and can significantly influence expression level measurements. Our approach easily allows for estimation and control of the “cellular detection rate” (CDR) while simultaneously estimating treatment effects.

Previously, Kharchenko et al. [6] developed a so-called three-component mixture model to test for differential gene expression while accounting for bimodal expression. Their approach is limited to two-class comparisons and cannot adjust for important biological covariates such as multiple treatment groups and technical factors such as batch or time information, limiting its utility in more complex experimental designs. Several methods have been proposed for modeling bulk RNA-seq data that permit sophisticated modeling through linear [12] or generalized linear models [13, 14], but these models have not yet been adapted to single-cell data because they do not properly account for the observed bimodality in expression levels. This is particularly important when adjusting for covariates that might affect the expression rates. As we will demonstrate later, such model mis-specification can significantly affect sensitivity and specificity when detecting differentially expressed genes and gene sets.

**Fig. 1:**
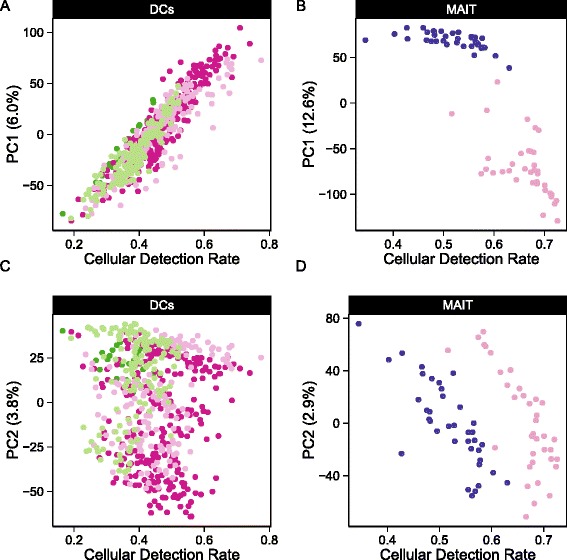
Cellular detection rate correlates with the first two principal components of variation. The fraction of genes expressed, or cellular detection rate (CDR) correlates mostly with the **a**,**c**) first principal component (PC) of variation in the myeloid dendritic cells (DC) data set and mostly with the second PC in the **b**,**d**) mucosal-associated invariant T (MAIT) data set

Here, we propose a hurdle model tailored to the analysis of scRNA-seq data, providing a mechanism to address the challenges noted above. It is a two-part generalized linear model that simultaneously models the rate of expression over the background of various transcripts, and the positive expression mean. Leveraging the established theory for generalized linear modeling allows us to accommodate complex experimental designs while controlling for covariates (including technical factors) in both the discrete and continuous parts of the model. We introduce the CDR: the fraction of genes that are detectably expressed in each cell. As discussed above, this acts as a proxy for both technical (e.g., dropout, amplification efficiency) and biological factors (e.g., cell volume and extrinsic factors other than treatment of interest) that globally influence gene expression. As a result, it represents an important source of variability in scRNA-seq data that needs to be modeled (Fig. 1). Our approach of modeling the CDR as a covariate offers an alternative to the weight correction of Shalek et al. [5] that does not depend on the use of control genes and allows us to jointly estimate nuisance and treatment effects. Our framework permits the analysis of complex experiments, such as repeated single-cell measurements under various treatments or longitudinal sampling of single cells from multiple subjects with a variety of background characteristics (e.g., sex, age), because it can easily be extended to accommodate random effects. These features are especially important when sampling single cells because there are multiple sources of variance (e.g., cell-to-cell variance within a subject, and subject-to-subject variance). These type of experiments and designs will become routine in future single-cell studies, such as for clinical trials where single-cell assays will be performed on large cohorts with complex designs.

In our hurdle model, differences between treatment groups are summarized with pairs of regression coefficients whose sampling distributions are available through bootstrap or asymptotic expressions, enabling us to perform complementary differential gene expression and gene set enrichment analyses (GSEA). We use an empirical Bayesian framework to regularize model parameters, which helps improve inference for genes with sparse expression, much like what has been done for bulk gene expression [15]. Our GSEA approach accounts for gene–gene correlations, which is important for proper control of type I errors [16]. This GSEA framework is particularly useful for synthesizing observed gene-level differences into statements about pathways or modules. Finally, our model yields “single cell residuals” that can be manipulated to interrogate cellular heterogeneity and gene–gene correlations across cells and conditions. We have named our approach MAST for “Model-based Analysis of Single-cell Transcriptomics.”

We illustrate the method on two data sets. We first apply our approach to an experiment comparing primary human non-stimulated and cytokine-activated mucosal-associated invariant T (MAIT) cells. MAST identifies novel expression signatures of activation, and the single-cell residuals produced by the model highlight a population of MAIT cells showing partial activation but no induction of effector function. We then illustrate the application of MAST to a previously published complex experiment studying temporal changes in murine bone marrow-derived dendritic cells subjected to lipopolysaccharide (LPS) stimulation. We both recapitulate the findings of the original publication and describe additional coordinated gene expression changes at the single-cell level across time in LPS-stimulated myeloid dendritic cells (mDC).

## Results and discussion

Our MAST framework models single-cell gene expression using a two-part generalized linear model. One component of MAST models the discrete expression rate of each gene across cells, while the other component models the conditional continuous expression level (conditional on the gene being expressed). We define the CDR as the proportion of genes expressed in a single cell.

The CDR for cell *i* is:

1 $$ CD{R}_i=1/N{\displaystyle \sum_{g=1}^N}{z}_{ig} $$

where *z*_*ig*_ is an indicator if gene *g* in cell *i* was expressed above *background*. We consider the implications of setting the background to zero, or alternately to a conservatively estimated non-zero threshold (see Additional file 1: Methods). The CDR is not sensitive to how the background is defined, nor does it change substantially when only putative control (housekeeping) genes are used in the summation in equation 1. Our thresholding approach does not adversely affect detection of differentially expressed genes and serves to make the continuous expression (Et > 0) more normal (Additional file 1: Figure S1).

### MAST can account for variation in the cellular detection rate

The principal component analysis (PCA) shown in Fig. 1 demonstrates that the CDR (see “Methods”) is an important source of variability. It correlates strongly with the second principal component (PC, Pearson’s rho = 0.76 grouped, 0.91 stimulated, 0.97 non-stimulated) in the MAIT data set and with the first PC (rho = 0.92 grouped, 0.97 non-stimulated, 0.92 LPS (lipopolysaccharide), 0.89 PAM (synthetic triacylated lipopeptide), 0.92 PIC (viral-like double-stranded RNA)) in the mDC data set. Given that we observe larger CDR variability within treatment groups than across groups, it is likely that the CDR is a nuisance factor. This is further supported by the fact that the CDR calculated using control (e.g., housekeeping) genes was highly correlated with the CDR calculated over all genes (Additional file 1: Figure S2). Its role as a principal source of variation persisted across experiments (Figure 1).

We thus conjecture that CDR is a proxy for unobserved nuisance factors that should be explicitly modeled. In particular, we suggest that the CDR captures variation in global transcription rates due to differences in cell size (among other factors) [11], as well as technical variation due to factors such as cell viability and efficiency in first strand synthesis. Fortunately, MAST easily accommodates covariates, such as the CDR, and more importantly allows joint, additive modeling of them with other biological variables of interest, with the effect of each covariate decomposed into its discrete and continuous parts. Applying an analysis of deviance with MAST (see “Methods”), we quantified the amount of variability that could be attributed to CDR. The CDR accounted for 5.2 % of the deviance in the MAIT data set and 4.8 % in the mDC data set for the average gene, and often much more than that: it comprised more than 9 % of the deviance in over 10 % of genes in both data sets, particularly for the discrete component of the model (Additional file 1: Figure S3). It should also be noted that the CDR deviance estimates for many of the genes were comparable to (if not greater than) the treatment deviance estimates. It is possible that the CDR and treatment effects could be partially confounded, for example, treated cells could become larger in volume. We explored the effect of confounding between the CDR and treatment effects on the MAST false positive rate in the presence and absence of CDR control in the MAST model (Additional file 1: Figure S4A, B). Controlling for CDR improved the sensitivity and specificity of MAST in the presence of confounding, and did not negatively impact its performance either in the absence of confounding or in the absence of a CDR effect.

That CDR predicts expression levels contradicts the model of independent expression between genes, because the level of expression (averaged across many genes) would not affect the level in any given gene were expression independent. It is especially important to adjust for CDR when testing for co-expression between genes, or the apparent correlation between genes is greatly inflated (see “Residual analysis identifies networks of co-expressed genes implicated in MAIT cell activation”).

Finally, we investigated the relationship between our approach and the weight correction of Shalek et al. [5] and other technical bias correction approaches like Remove Unwanted Variation (RUV) and Surrogate Variable Analysis (SVA; Additional file 1: Figure S5A, B) [17, 18]. We observed a strong linear relationship between the CDR and the weights of Shalek et al. [5], as well as with the first component of SVA and second component of RUV. Thus, use of the CDR as a covariate can be seen as a statistically rigorous way to correct for the dropout biases of Shalek et al. [5], without the need to use control genes. More importantly, it provides the ability to control for these biases while estimating treatment effects. Although CDR was correlated to the latent components found via RUV or SVA in the data sets we consider here (Additional file 1: Figure S5C), CDR has the advantage of biological interpretability as a cellular scaling factor.

### Single-cell sequencing identifies a transcriptional profile of MAIT cell activation

We applied MAST to our MAIT data set to identify genes up-regulated or down-regulated by cytokine stimulation while accounting for variation in the CDR (see “Methods”). We detected 291 differentially expressed genes, as opposed to 1413 when excluding CDR. To determine whether this was due to a change in ranking or simply a shift in significance, we compared the overlap between the top *n* genes in both models (varying *n* from 100 to 1413), and found that, on average, 35 % (range 32–38 %) of genes were excluded when CDR was modeled, suggesting that inclusion of this variable allows global changes in expression, manifest in the CDR, to be decomposed from local changes in expression. This was supported by gene ontology (GO) enrichment analysis (Additional file 1: Figure S6) of these CDR-specific genes (n = 539), where we saw no enrichment for modules associated with the treatment of interest. These CDR-specific GO terms (e.g., involvement of regulation of RNA stability and protein folding) may hint at the biology underlying differences in the CDR that are not necessarily associated with treatment.

In order to assess the type-I error rate of our approach, we also applied MAST to identify differentially expressed genes across random splits of the MAIT cells. As expected, MAST did not detect any significant differences (Additional file 1: Figure S7A ,B), whereas DEseq and edgeR, designed for bulk RNA-seq, detected a large number of differentially expressed genes even at a stringent nominal false discovery rate (FDR). SCDE, a single-cell RNA-seq specific method, also had higher FDRs than MAST. Permutation analysis demonstrated that the null distribution of the MAST test statistic was well calibrated (Additional file 1: Figure S8A).

We examined the GO enrichment of genes detected by limma, edgeR, DESeq, or SCDE but not MAST and found that these sets generally lacked significant enrichment for modules related to the treatment of interest (Additional file 1: Figures S9–S12). MAST with CDR control also detected enrichment of immune-specific GO terms at a higher rate than other methods (Additional file 1: Figure S13). MAST’s testing framework has better sensitivity and specificity than these approaches. Among models that do not adjust for CDR, SCDE performs relatively well but trails MAST and limma, which can adjust for CDR.

**Fig. 2:**
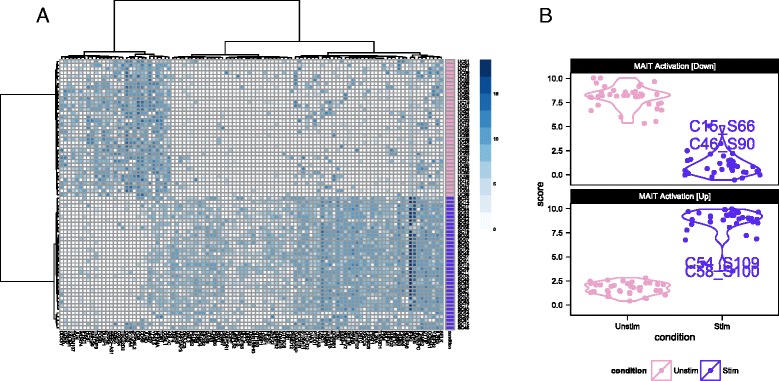
Single-cell expression (log_2_-transcripts per million) of the top 100 genes identified as differentially expressed between cytokine (IL18, IL15, IL12)-stimulated (*purple*) and non-stimulated (*pink*) MAIT cells using MAST (**a**). Partial residuals for up-regulated and down- regulated genes are accumulated to yield an activation score (**b**), and this score suggests that the stimulated cells have a more heterogeneous response to stimulation than do the non-stimulated cells

Figure 2a shows the single-cell expression (log_2_-transcripts per million [TPM]) of the top 100 genes identified as differentially expressed between cytokine (IL18, IL15, IL12)-stimulated and non-stimulated MAIT cells using MAST. Following stimulation with IL12, IL15, or IL18, we observed increased expression in genes with effector function, including interferon-*γ* (*IFNG*), granzyme-B (*GZMB*) and a concomitant down-regulation of the AP-1 transcription factor network. Up-regulation of IFNG and GZMB following cytokine stimulation has also been reported in natural killer (NK), natural killer T-cells (NKT), and memory T cells. CD69 is an early and only transient marker of activation that can be induced by stimulation of the T cell receptor or by cytokine signals. Its down-regulation at the mRNA level after 24 h likely precedes subsequent protein-level down-regulation [19–21].

We used these lists of up-regulated and down-regulated genes to define a MAIT activation score that differentiates between stimulated and non-stimulated MAITs as shown in Fig. 2b. This yields a score for each cell, based on the model fit and adjusting for nuisance factors (see “Methods”), defined as the expected expression level across genes in a module. The score differentiates stimulated and non-stimulated cells, and demonstrates that the stimulated MAIT population was more heterogeneous in its expression phenotype. In particular, a few stimulated MAIT cells (SC08, SC54, SC48, SC15, SC46, and SC61 in Fig. 2a) exhibited low expression of *IFNG* response genes, suggesting these cells did not fully activate despite stimulation. Post-sort experiments via flow cytometry showed that the sorted populations were over 99 % pure MAITs (Additional file 1: Figure S14A), exhibited a change in cell size upon stimulation (Additional file 1: Figure S14B), and that up to 44 % of stimulated MAITs did not express *IFNG* or *GZMB* following cytokine stimulation (Additional file 1: Figure S14C). The non-responding cells in the RNA-seq experiment likely correspond to these non-responding cells from the flow cytometry experiment, and the observed frequencies of these cells in the RNA-seq and flow populations are consistent with each other (probability of observing 6 or fewer non-responding cells = 0.16 under binomial sampling). We discuss this heterogeneity in a further section. Importantly, the lists of up-regulated and down-regulated genes can be used to define gene sets for GSEA in order to identify transcriptional changes related to MAIT activation in bulk experiments.

### GSEA highlights pathways implicated in MAIT cell activation

**Fig. 3:**
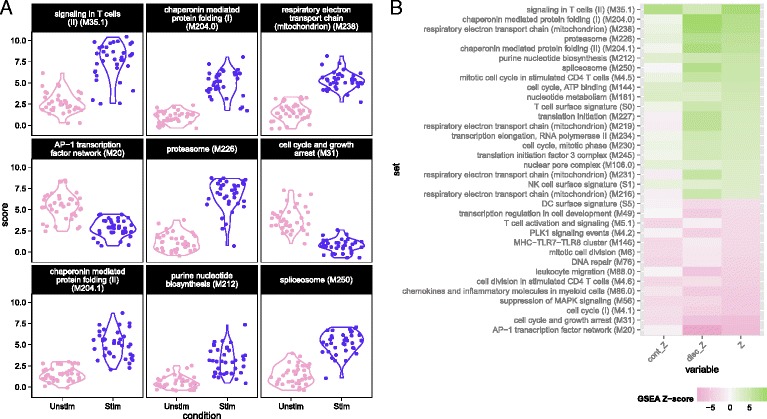
Module scores for individual cells for the top nine enriched modules (**a**) and decomposed Z-scores (**b**) for single-cell gene set enrichment analysis in the MAIT data set, using the blood transcription modules (BTM) database. The distribution of module scores suggests heterogeneity among individual cells with respect to different biological processes. Enrichment of modules in stimulated and non-stimulated cells is due to a combination of differences in the discrete (proportion) and continuous (mean conditional expression) components of genes in modules. The combined Z-score reflects the enrichment due to differences in the continuous and discrete components

We used MAST to perform GSEA (see “Methods”) in the MAIT data using the blood transcriptional modules of Li et al. [22]. The cell-level scores for the top nine enriched modules (Fig. 3a) continued to show significant heterogeneity in the stimulated and non-stimulated cells, particularly for modules related to T-cell signaling, protein folding, proteasome function, and the AP-1 transcription factor network. Although the standard deviations of the module scores were greater for stimulated than non-stimulated cells in seven of the top nine enriched modules (Additional file 1: Table S2), the magnitude of variability for stimulated and non-stimulated cells was fairly similar. Enrichment in stimulated cells and non-stimulated cells is displayed for each module for the discrete and continuous components of the model in Fig. 3b (see “Methods”), as well as a Z-score combining the discrete and continuous parts. The enrichment in the T-cell signaling module was driven by the increased expression of *IFNG*, *GZMB*, *IL2RA*, *IL2RB*, and *TNFRSF9*, five of the six genes in the module. Stimulated cells also exhibited increased energy usage, translation, and protein synthesis, while down-regulating genes were involved in cell cycle growth and arrest (and other cell cycle related modules). The down-regulation of cell cycle growth inhibition genes indicates that IL12, IL15, and IL18 signals are sufficient to prepare MAIT cells for cell proliferation. Interestingly, we observed down-regulation of mRNA transcripts from genes in the AP-1 transcription factor network. This has been previously described in dendritic cells in response to LPS stimulation [23] and, indeed, we observed this effect in the mDC data set analyzed here (Additional file 1: Fig. S15).

Our GSEA approach is more powerful than existing methods for bulk RNA-seq data (Additional file 1: Figure S16), and we discovered significantly enriched modules with clear patterns of stimulation-induced changes that other methods omit (Additional file 1: Figure S17). Two such modules include the “T-cell surface signature” and “chaperonin mediated protein folding,” whose component genes showed elevated expression in response to stimulation (Additional file 1: Fig. S17A–D). These additional discoveries are not solely due to greater permissiveness in MAST. We applied MAST to identify differentially expressed gene sets across random partitions of the non-stimulated cells, to examine its FDR. As expected, MAST did not detect any significant differences, which suggests that it has good type I error control (Additional file 1: Figure S7A).

### Residual analysis identifies networks of co-expressed genes implicated in MAIT cell activation

**Fig. 4:**
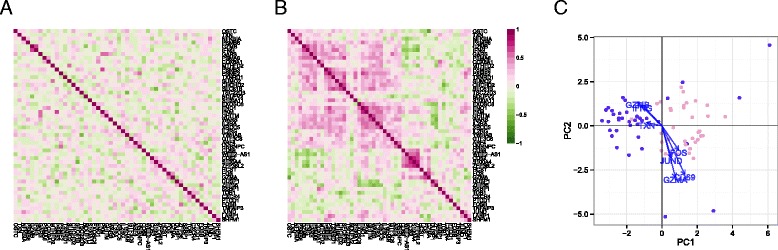
Gene–gene correlation (Pearson’s rho) of model residuals in non-stimulated (**a**) and stimulated (**b**) cells, and a principal components analysis biplot of model residuals (**c**) on both populations using the top 50 marginally differentially expressed genes. As marginal changes in the genes attributable to stimulation and CDR have been removed, clustering of subpopulations in (c) indicates co-expression of the indicated genes on a cellular basis. *PC* principal component

Much of the heterogeneity between the non-responding and responding stimulated cells remained even after removal of marginal (gene-level) stimulation effects. Given that MAST models the expected expression value for each cell, we can compute residuals adjusted for known sources of variability (see “Methods”). The residuals can be compared across genes to characterize cellular heterogeneity and correlation. We observed co-expression in the residuals from stimulated cells that was not evident in the non-stimulated group (Fig. 4a, b). Because the residuals removed any marginal changes due to stimulation in each gene, the average residual in the two groups is comparable. The co-expression observed, meanwhile, is due to individual cells expressing these genes dependently, where pairs of genes appear together more often than expected under a model of independent expression.

Two clusters of co-expressed genes stood out in the residuals of the stimulated cells (Fig. 4b). These clusters showed coordinated, early up-regulation of *GZMB* and *IFN-*γ in response to stimulation in MAIT cells and a concomitant decrease in *CD69* expression, an early and transient activation marker. PCA of the model residuals highlighted the non-responsive stimulated MAIT cells (Fig. 4c).

Accounting for the CDR reduced the background correlation observed between genes (Additional file 1: Figure S18), with nearly 25 % of pairwise correlations decreasing after CDR correction. When the CDR was included in the model, the number of differentially expressed genes with significant correlations across cells (FDR adjusted *p*-value < 1 %) decreased from 73 to 61 in the stimulated cells, and from 808 to 15 in non-stimulated cells. This shows that adjusting for CDR is also important for co-expression analyses because it reduces background co-expression attributable to cell volume, which otherwise results in dense, un-interpretable gene networks.

### Residual analysis of MAIT non-responding stimulated cells

The hurdle model expression residuals identified six MAIT cells that did not have a typical activated expression profile in response to stimulation (Figs 2 and 3). The proportion of these cells detected in the scRNA-seq experiment was consistent with what was detected in the flow cytometry experiment. The cells exhibited lower levels of *IFNG* and *GZMB* than activated cells (Additional file 1: Figure S19A), but also exhibited decreased expression of AP-1 component genes *FOS* and *FOSB*, consistent with other stimulated cells (Additional file 1: Figure S19B). They did not produce *IFNG* or *GZMB* upon cytokine stimulation and exhibited expression profiles intermediate to non-stimulated and stimulated cells (Additional file 1: Figure S19C).

### Temporal expression patterns of mouse dendritic cell maturation

Shalek et al. [5] analyzed murine bone marrow-derived dendritic cells simulated using three pathogenic components over the course of 6 h and estimated the proportion of cells that expressed a gene and the expression level of expressing cells. We compared results from applying our model to those obtained by Shalek et al. [5] when analyzing their LPS-stimulated cells. As with the MAIT analysis, we used MAST adjusting for the CDR. MAST identified a total of 1359 differentially expressed genes (1996 omitting the CDR), and the CDR accounted for 5.2 % of the model deviance in the average gene.

The most significantly elevated genes at 6 h included *CCL5*, *CD40*, *IL12B*, and interferon-inducible (*IFIT*) gene family members, while down-regulation was observed for *EGR1* and *EGR2*, transcription factors that are known to negatively regulate dendritic cell immunogenicity [24].

### GSEA of mouse bone marrow-derived dendritic cells

**Fig. 5:**
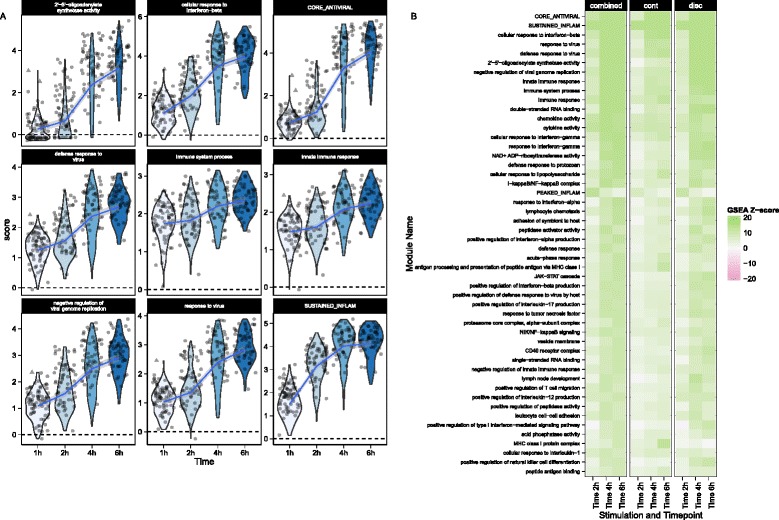
Module scores (**a**) and decomposed Z-scores (**b**) for single-cell gene set enrichment analysis for lipopolysaccharide (LPS)-stimulated myeloid dendritic cells (mDC data set), using the mouse gene ontology (GO) biological process database. The change in single-cell module scores over time for the nine most significantly enriched modules in response to LPS stimulation are shown in (**a**). The “core antiviral”, “peaked inflammatory,” and “sustained inflammatory” modules are among the top enriched modules, consistent with the original publication. Additionally, we identified the GO modules “cellular response to interferon-beta” and “response to virus,” which behave analogously to the core antiviral and sustained inflammatory modules. No GO analog for the “peaked inflammatory” module was detected. The majority of modules detected exhibited enrichment relative to the 1 h time point (thus increasing with time). The “early marcher” cells identified in the original publication are highlighted here with *triangles*. We show the top 50 most significant modules (**b**). The combined Z-score summarizes the changes in the discrete and continuous components of expression

We performed GSEA with the mouse GO modules and three modules identified by Shalek et al. [5]. The blood transcriptional modules of Li et al. [22] are shown in Additional file 1: Figure S15. Figure 5 shows module scores for significant GSEA modules for the LPS-stimulated cells where the heatmap represents Z values (see “Methods” for details). Besides finding signatures consistent with the modules from Shalek et al. (Fig. 5a), we identified modules that showed similar annotation and overlap, significantly with the “core antiviral” and “sustained inflammatory” signatures, including several modules linked to type 1 interferon response and antiviral signatures (Fig. 5b). The “cellular response to interferon-beta” signature (n = 22) overlapped with the original core antiviral signature (n = 99) by 13 genes (hypergeometric *p* = 1.24 × 10^−23^). The “response” and “defense response to virus” signatures overlapped with the core antiviral signature by 17 of 43 and 22 of 74 genes (hypergeometric *p* = 3.64 × 10^−26^ and 4.08 × 10^−29^, respectively), suggesting the core antiviral signature captures elements of these known signatures. The “chemokine” (n = 16) and “cytokine activity” (n = 51) modules overlapped with the sustained inflammatory (n = 95) module by 5 and 12 genes, respectively (hypergeometric *p* = 5.10 × 10^−9^ and 9.53 × 10^−16^). Our modeling approach identified the two “early marcher” cells in the core antiviral module (marked with triangles on Fig. 5a) corresponding to the same cells highlighted in figure 4b of Shalek et al. [5]. Other modules exhibiting significant time-dependent trends included a module of genes involved in the AP-1 transcription factor network that was down-regulated (Additional file 1: Figure S15), a finding which has been previously shown in human monocytes following LPS stimulation [23]. As with the MAITs, GSEA permutation analysis to evaluate type I error rates did not identify any significant modules (data not shown). These results further confirm the original findings and demonstrate the increased sensitivity of our approach. GSEA heatmaps for the other stimulations can be found in Additional file 1: Figure S20.

### Residual analysis of mouse bone marrow-derived dendritic cells identifies sets of co-expressed genes

**Fig. 6:**
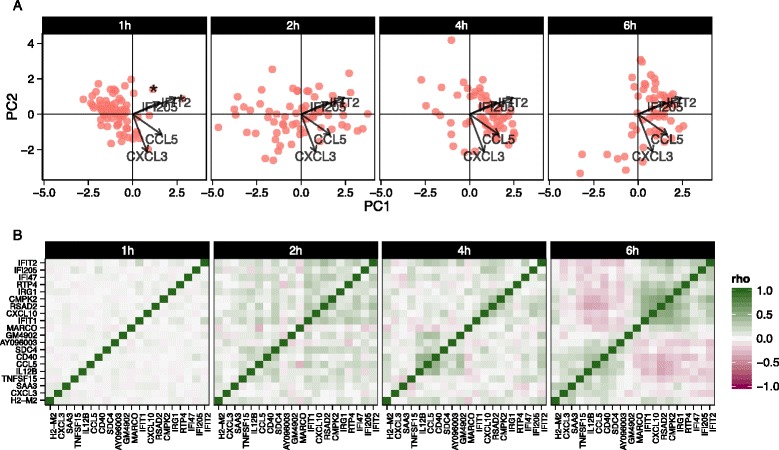
Principal components analysis biplot of model residuals (**a**) and gene–gene correlation (Pearson’s rho) of model residuals (**b**) by time point for lipopolysaccharide-induced myeloid dendritic cells (mDC data set) using 20 genes with the largest log-fold changes, given significant (false discovery rate q < 0.01) marginal changes in expression. Principle component 1 (*PC1*) is correlated with change over time. The two “early marcher” cells are highlighted by an *asterisk* at the 1 h time point. Correlation structure in the residuals is increasingly evident over time and can be clearly observed at the 6 h time point compared to the earlier time points

We also explored stimulation-driven correlation patterns. PCA (Fig. 6a) of the model residuals demonstrated a clear time trend associated with PC1, as cells increase co-expression of interferon-activated genes. After removing the marginal stimulation and adjusting for the CDR, we observed correlation between chemokines *CCL5*, TNF receptor *CD40*, and *IFIT* genes (Fig. 6b). A principal finding of the original publication was the identification of a subset of cells that exhibited an early temporal response to LPS stimulation. Recapitulating the original results here, when we examine the PCA of the residuals using the genes in the core antiviral module, we can identify the “early marcher” cells at the 1 h time-point (Additional file 1: Figure S21). The co-expression plot for other stimulations can be found in the supplementary material (Additional file 1: Figures S22 and 23).

## Conclusion

We have presented MAST, a flexible statistical framework for the analysis of scRNA-seq data. MAST is suitable for supervised analyses about differential expression of genes and gene modules, as well as unsupervised analyses of model residuals, to generate hypotheses regarding co-expression of genes. MAST accounts for the bimodality of single-cell data by jointly modeling rates of expression (discrete) and positive mean expression (continuous) values. Information from the discrete and continuous parts is combined to infer changes in expression levels using gene or gene set-based statistics. Because our approach uses a generalized linear framework, it can be used to jointly estimate nuisance variation from biological and technical sources, as well as biological effects of interest. In particular, we have shown that it is important to control for the proportion of genes detected in each cell, which we refer to as the CDR, because this factor can single-handedly explain 13 % of the variability in the 90 % percentile gene. Adjusting for CDR at least partially controls for differences in abundance due to cell size and other extrinsic biological and technical effects. Using several scRNA-seq data sets, we showed that our approach provides a statistically rigorous improvement to methods proposed by other groups in this context [5]. Although MAST has greatest efficiency when the continuous (log)-expression is normally distributed, transformations (such as the Box-Cox) could also be applied if the non-zero continuous measurements are skewed.

As discussed by Padovan-Merhar et al. [11], care must be taken when interpreting experiments where the system shows global changes in CDR across treatment groups. The question is essentially ontological: is the CDR a mediator of the treatment effect (is it caused by the treatment and intermediate to expression of the gene of interest), or does it confound the treatment effect (does it happen to co-occur with treatment)? Regardless, the CDR-adjusted treatment estimates are interpreted as the change in expression due to treatment, if CDR were held constant between the two conditions.

Two other alternative uses of the CDR are of note. It is also possible to use CDR as a precision variable (an uncorrelated secondary cause) by centering the CDR within each treatment groups, which makes the CDR measurement orthogonal to treatment. This would implicitly assume that the observed changes are treatment induced, while still modeling the heterogeneity in cell volume within each treatment group. An alternative approach would be to estimate the CDR coefficient using a set of control genes assumed to be treatment invariant, such as housekeeping or ERCC spike-ins [25, 26] and including it as an offset to the linear predictors in the regression. An analogous approach is undertaken by Buettner et. al. [26]. As noted by Hicks et al*.* [27], the optimal approach to handle confounding between technical and biological effects on the CDR is to design experiments with biological replicates across multiple batches. Finally, we note that while the methodology presented here was developed using scRNA-seq data sets, it appears applicable to other single-cell gene expression platforms where bimodal, conditionally normal expression patterns are seen such as single-cell RNA-seq with unique molecular identifiers.

## Methods

### Data sets

Data for the MAIT study were derived from a single donor who provided written informed consent for immune response exploratory analyses. The study was approved by the Fred Hutchinson Cancer Research Center institutional review board.

### MAIT cell isolation and stimulation

Cryopreserved peripheral blood mononuclear cells were thawed and stained with Aqua Live/Dead Fixable Dead Cell Stain and the following antibodies: CD3, CD8, CD4, CD161, Vα7.2, CD56, and CD16. CD8^+^ MAIT cells were sorted as live CD3^+^CD8^+^ CD4^-^CD161^hi^Vα7.2^+^ cells and purity was confirmed by post-sort fluorescence-activated cell sorting analysis. Sorted MAIT cells were divided into aliquots and immediately processed on a C1 Fluidigm (Fluidigm, South San Francisco, CA) machine or treated with a combination of IL-12 (eBioscience, San Diego, CA), IL-15 (eBioscience), and IL-18 (MBL, Worburn, MA ) at 100 ng/mL for 24 h followed by C1 processing.

### C1 processing, sequencing, and alignment

After flow sorting, single cells were captured on the Fluidigm C1 Single-Cell Auto Prep System (C1), lysed on chip, and subjected to reverse transcription and cDNA amplification using the SMARTer Ultra Low Input RNA Kit for C1 System (Clontech, Mountain View, CA). Sequencing libraries were prepared using the Nextera XT DNA Library Preparation Kit (Illumina, San Diego, CA) according to C1 protocols (Fluidigm). Barcoded libraries were pooled and quantified using a Qubit Fluorometer (Thermo Scientific Life Technologies, Grand Island, NY). Single-read sequencing of the pooled libraries was carried out either on a HiScanSQ or a HiSeq2500 sequencer (Illumina) with 100-base reads, using TruSeq v3 Cluster and SBS kits (Illumina) with a target depth of >2.5 M reads. Sequences were aligned to the UCSC Human Genome Assembly version 19, gene expression levels quantified using RSEM [28], and TPM values loaded into R [29] for analyses. See Additional file 1 for more details on data processing procedures.

### Time-series stimulation of mouse bone-marrow derived dendritic cells

Processed RNA-seq data (TPM) were downloaded from the Gene Expression Omnibus [GEO: GSE41265]. Alignment, pre-processing, and filtering steps have been previously described [5]. Low quality cells were filtered as described in Shalek et al. [5].

### Single-cell RNA-seq hurdle model

We model the log_2_(TPM + 1) expression matrix as a two-part generalized regression model. The gene expression rate was modeled using logistic regression and, conditioning on a cell expressing the gene, the expression level was modeled as Gaussian.

Given normalized, possibly thresholded (see Additional file 1), scRNA-seq expression *Y* = [*y*_*ig*_], the rate of expression and the level of expression for the expressed cells are modeled conditionally independent for each gene *g*. Define the indicator Z = [*z*_*ig*_], indicating whether gene *g* is expressed in cell *i* (i.e., *z*_*ig*_ = 0 if *y*_*ig*_ = 0 and *z*_*ig*_ = 1 if *y*_*ig*_ > 0). We fit logistic regression models for the discrete variable *Z* and a Gaussian linear model for the continuous variable (*Y* | *Z* = 1) independently, as follows:

$$ logit\left( Pr\left({Z}_{ig}=1\right)\right)={\mathrm{X}}_{\mathrm{i}}{\upbeta}_{\mathrm{g}}^{\mathrm{D}} $$

$$ \Pr \left({Y}_{ig}=y\Big|{Z}_{ig} = 1\right) = \mathrm{N}\left({\mathrm{X}}_{\mathrm{i}}{\upbeta}_{\mathrm{g}}^{\mathrm{C}},\ {\sigma}_g^2\right) $$

The regression coefficients of the discrete component are regularized using a Bayesian approach as implemented in the *bayesglm* function of the *arm* R package, which uses weakly informative priors [30] to provide sensible estimates under linear separation (See Additional file 1 for details). We also perform regularization of the continuous model variance parameter, as described below, which helps to increase the robustness of gene-level differential expression analysis when a gene is only expressed in a few cells.

We define the CDR as the proportion of genes detected in each cell. The CDR for cell *i* is:

$$ CD{R}_i=1/N{\displaystyle \sum_{g=1}^N}{z}_{ig} $$

An advantage of our approach is that it is straightforward to account for CDR variability by adding the variable as a covariate in the discrete and continuous models (column of the design matrix, *X*, defined above). In the context of our hurdle model, inclusion of the CDR covariate can be thought of as the discrete analog of global normalization, and as we show in the examples, this normalization yields more interpretable results and helps decrease background correlation between genes, which is desirable for detecting genuine gene co-expression.

### Shrinkage of the continuous variance

As the number of expressed cells varies from gene to gene, so does the amount of information available to estimate the residual variance of the gene. However, many genes can be expected to have similar variances. To accommodate this feature of the assay, we shrink the gene-specific variance estimates to a global estimate of the variance using an empirical Bayes method. Let *τ*_*g*_^2^ be the precision (1/variance) for *Y*_*g*_|*Z*_*g*_ = 1 in gene g. We suppose *τ*_*g*_^2^ ∼ *Gamma*(*α*, *β*), find the joint likelihood (across genes), and integrate out the gene-specific inverse variances. Then the maximum likelihood is used to estimate *α* and *β*. Owing to conjugacy, these parameters are interpretable, providing 2*α* pseudo-observations with precision *β*/*α*. This leads to a simple procedure where the shrunken gene-specific precision is a convex combination of its maximum likelihood estimate (MLE) and the common precision. This approach accounts for the fact that the number of cells expressing a gene varies from gene to gene. Genes with fewer expressed cells end up with proportionally stronger shrinkage, as the ratio of pseudo observations to actual observations is greater. Further details are available in Additional file 1.

### Testing for differential expression

Because *Z*_*g*_ and *Y*_*g*_ are defined conditionally independent for each gene, tests with asymptotic *χ*^2^ null distributions, such as the likelihood ratio or Wald tests, can be summed and remain asymptotically *χ*^2^, with the degrees of freedom of the component tests added. For the continuous part, we used the shrunken variance estimates derived through our empirical Bayes approach described above. The test results across genes can be combined and adjusted for multiplicity using the FDR adjustment [31]. In this paper, we declare a gene differentially expressed if the FDR adjusted *p*-value is less than 0.01 and the estimated fold-change is greater than 1.5 (on a log_2_ scale).

### Gene set enrichment analysis

Our competitive GSEA compares the average model coefficient in the test set (gene set of interest) to the average model coefficient in the null set (everything else) with a Z-test. Suppose the genes are sorted so that the first *G*_0_ genes are in the null set, and the last *G* − *G*_0_ genes are in the test set. Then, for example, to test the continuous coefficients in the gene set, the sample means of the coefficients in the test and null sets are calculated, that is, calculate: $\widehat{\theta}=1/\left(G-{G}_0\right){\displaystyle {\sum}_{g={G}_0+1}^G{\widehat{\beta}}_g}$ and $\widehat{\theta_0}=1/{G}_0\kern0.5em {\displaystyle {\sum}_{g=1}^{G_0}{\widehat{\beta}}_g}$. The sampling variance of ${\widehat{\theta}}_0$, in principle, is equal to $1/{G}_0\left({\displaystyle {\sum}_{g=1}^nVar\left({\widehat{\beta}}_g\right)+2{\displaystyle {\sum}_{1\le g<h<{G}_0}Cov\left({\widehat{\beta}}_g,{\widehat{\beta}}_h\right)}}\right)$, and similarly for $\widehat{\theta}$.

Given this sampling variance, a Z test can be formed by comparing $Z=\frac{\widehat{\theta}-{\widehat{\theta}}_0}{\sqrt{\widehat{Var}\left(\widehat{\theta}\right)+\widehat{Var}\left({\widehat{\theta}}_0\ \right)}}$.

We estimate $Var\left({\widehat{\beta}}_g\ \right)$ and $Cov\left(\ {\widehat{\beta}}_g,\ {\widehat{\beta}}_h\ \right)$ via bootstrap, to avoid relying on asymptotic approximations. In practice, we found only a few (<100) bootstrap replicates were necessary to provide stable variance-covariance estimates; however, even this modest requirement can be relaxed for exploratory analysis by assuming independence across genes and using model-based (asymptotic) estimates.

Z scores are formed and calculated equivalently for the logistic regression coefficients. GSEA tests are done separately on the two components of the hurdle model and the results from the two components are combined using Stouffer’s method [32], which favors consensus in the two components [33] (see Additional file 1 for details). The approach is similar to that used by CAMERA [16] for bulk experiments in its accounting for inter-gene correlation that is known to inflate the false significance (type-I error) in permutation-based GSEA protocols [16], although it differs in that it uses the sampling variance of each model coefficient to estimate the variance of the average coefficient, whereas CAMERA uses the empirical variance of the model coefficients. In our analyses, we used the Emory blood transcriptional modules [22] as well as mouse GO annotations available from the Mouse Genome Informatics website [34].

### GO enrichment analysis

Testing for enriched GO terms based on a list of genes was performed with the GOrilla online tool by comparing an unranked target list against a background list [35].

### Residual analysis

The hurdle model, in general, provides two residuals: one for the discrete component and one for the continuous component. Standardized deviance residuals are calculated for the discrete and continuous component separately, and then we combine the residuals by averaging them. If a cell is unexpressed, then its residual is missing and it is omitted from the average (details in Additional file 1: Methods).

### Module scores

In order to assess the degree to which each cell exhibits enrichment for each gene module, we use quantities available through our model to define module “scores,” which are defined as the observed expression corrected for CDR effect, analogous to those defined by Shalek et al. [5]. The score *s*_*ij*_ for cell *i* and gene *j* is defined as the observed expression corrected for the CDR effect: *s*_*ij*_ = *y*_*ij*_ − *ỹ*_*ij*_ where *ỹ*_*ij*_ is the predicted effect from the fitted model that excludes the treatment effects of interest. This can be interpreted as correcting the observed expression of gene *j* in cell *i* by subtracting the conditional expectation of nuisance effects. In our two part model, *ỹ*_*ij*_ = *ẑ*_*ij*_*ŷ*_*ij*_ , where *ẑ*_*ij*_ and *ŷ*_*ij*_ are the predicted values from the discrete and continuous components of our hurdle model.

A gene module score for cell *i* is the average of the scores for the genes contained in the module, that is, ∑_{*j* ∈ *module*}_*s*_*ij*_/|*module*|.

### Availability of supporting data

MAST is available as an R package (http://www.github.com/RGLab/MAST, doi: 10.5281/zenodo.18539), released under the GPL license. All data and results presented in this paper—including code to reproduce the results—are available at: (http://github.com/RGLab/MASTdata/archive/v1.0.1.tar.gz, doi: 10.5281/zenodo.19041). Raw data files have been submitted to NCBI’s sequence read archive under project accession SRP059458.

### Ethics

All experimental methods comply with the Helsinki Declaration.

## Additional file

Additional file 1:
**Supplemental methods, extended derivations, and supplementary figures and tables.** (PDF 3940 kb)

## Abbreviations

CDR: cellular detection rate
FDR: false discovery rate
GO: gene ontology
GSEA: gene set enrichment analysis
LPS: lipopolysaccharide
MAIT: Mucosal associated invariant T cells
MAST: Model-based analysis of single-cell transcriptomics
mDC: myeloid dendritic cells
NK: Natural Killer
NKT: Natural Killer T cells
PC: principal component
PCA: principal component analysis
scRNASeq: single-cell RNA sequencing
