# Supplementary material for: MAST: a flexible statistical framework for assessing transcriptional changes and characterizing heterogeneity in single-cell RNA sequencing data
Source: Genome Biol. 2015 Dec 10;16:278. doi: 10.1186/s13059-015-0844-5 (PMC4676162; doi:10.1186/s13059-015-0844-5)
Supplement: Additional file 1: — Supplemental methods, extended derivations, and supplementary figures and tables. (PDF 3940 kb) [file 13059_2015_844_MOESM1_ESM.pdf]

SUPPLEMENTAL METHODS AND EXTENDED DERIVATIONS

GREG FINAK, ANDREW MCDAVID, MASANAO YAJIMA, JINGYUAN DENG, VIVIAN GERSUK, ALEX SHALEK, CHLOE K. SCHLICTER, HANNAH W. MILLER, M. JULIANNA MCELRATH, MARTIN PRLIC, PETER S. LINSLEY, RAPHAEL GOTTARDO.

1. Data processing

1.1. **MAIT single-cell sequencing data processing.** Adaptor sequences from the FastQ files were trimmed by fastqtrimmer (Blankenberg et al., 2010). Sequences were aligned to the UCSC Human genome assembly version 19 and gene expression levels quantified using RSEM (Li and Dewey, 2011), and TPM values were loaded into R (Gentleman et al., 2004) for analyses. Libraries were deemed to be of good quality if they met the following conditions: exonic rate greater than 30%, percent of reads mapped to human greater than 60%, and number of genes with non-zero TPM values greater than 4000. Transcripts were annotated using the BioConductor (Gentleman et al., 2004) transcript annotation database `TxDB.Hsapiens.UCSC.hg19.knownGene`.

1.2. **Mouse dendritic cells (mDC).** The bone-marrow derived dendritic cells were processed and provided by the Shalek lab (see supplemental information on Shalek et al. (2014) for details). We removed the cluster disruptive cells using Lyz1 and Serpinb6b expression, as previously described.

For each dataset, an adaptive thresholding method was applied to groups of genes conditional on median expression level, to help discriminate background noise, with a minimum threshold of 1 $\log_2$-TPM (see section on thresholding). Following thresholding, invariant genes (expressed in fewer than 20% of cells) were removed. Throughout the paper we use thresholded $\log_2$TPM values. The thresholding procedure decreased the number of non-normally distributed genes in the mDC data set (conditional on Et > 0) from 602 to 241, and in the MAIT data set from 246 to 69, as determined by the Shapiro-Wilk test performed on the continuous component model residuals.

1.3. **Thresholding expression noise by adaptive gene pooling.** In previous studies, small, but non-zero expression values were thresholded using an arbitrary fixed threshold (Shalek et al., 2014). These conservative fixed thresholds do not allow any variation between genes for differing levels of background noise as suggested by Kharchenko et al. (2014). In order to adaptively determine the level of background noise, we propose a thresholding routine that shares information across genes. The N genes are divided into K equally spaced bins over the P cells based on each gene's median $\log_2$(count). The bins are indexed such that the median of bin k is greater than bin $k+1$, and so forth. This binning

allows for thresholding that varies with the expression level of the gene. For each bin we apply kernel density estimation and determine if the distribution is bimodal, then apply peak finding to estimate the threshold t_k as the minimum density point between the two major peaks in the bin. If the distribution for the kth bin is not bimodal, its threshold is set as follows. If the k^mth bin is the bin with the median threshold amongst bins where a reliable threshold could be found, we examine all bins $k*$ where $k* < k^m$ and set $t_{k*} = t_k^m$ if $t_{k*} > t_k^m$. Similarly, for all bins $k^{**} > k^m$, we set $t_{k^{**}} = t_k^m$ if $t_{k^{**}} < t_k^m$. This ensures that the thresholds are monotonically increasing and shares information across bins to impute thresholds for bins where the distribution of the data was not bimodal. This function is implemented in the `thresholdSCRNACountMatrix` function of the MAST package.

2. Model Specification

2.1. **Empirical Bayes derivation of variance hyper parameters.** Suppose there are genes $g = 1, \ldots, G$. Assume that the precision (inverse variance) for the continuous component of gene g is distributed

$$\tau_g|\alpha_0, \beta_0 \sim \text{Gamma-rate}(\alpha_0, \beta_0)$$

and that $i \neq j \Rightarrow \tau_i \perp \tau_j|\alpha_0, \beta_0$. Thus $\tau_g|\alpha_0, \beta_0$ has density

$$f(\tau_g|\alpha_0, \beta_0) = \tau_g^{\alpha_0 - 1} e^{-\tau_g \beta_0} \beta_0^{\alpha_0} / \Gamma(\alpha_0).$$

Assume that n_g cells have non-zero expression vector Y_g in gene g under the linear model $\mathrm{E}[Y_g|X] = X\eta$, with $\dim(\eta) = p$, so that

$$Y_g|\tau_g, \eta \sim \mathcal{N}(X\eta, \tau_g).$$

This implies that $R_g = \sum(y_i - \hat{\eta} X_i)^2$ is sufficient for τ_g and that statistic has scale chi-square distribution with $n_g - p$ degrees of freedom, or equivalently, a gamma-rate distribution with shape $\alpha_g = (n_g - p)/2$ and rate $\beta_g = \tau_g/2$. Here $\hat{\eta}$ is the typical OLS estimator.

The joint distribution of $\tau_g, R_g|\alpha_0, \beta_0$ has density

$$f(R_g, \tau_g|\alpha_0, \beta_0) = \tau_g^{\alpha' - 1} \exp\left(-\tau_g \beta'\right) \beta_0^{\alpha_0} / \Gamma(\alpha_0) R_g^{(n_g - p)/2 - 1} \left(1/2\right)^{(n_g - p)/2} / \Gamma\left((n_g - p)/2\right).$$

for $\alpha' = \alpha_0 + (n_g - p)/2$ and $\beta' = \beta_0 + R_g/2$. In terms of τ_g this density has the kernel of a gamma distribution, with aforementioned parameters, so that marginalizing out τ_g yields

$$\begin{aligned}
f(R_g|\alpha_0, \beta_0) &= \frac{\Gamma(\alpha')\beta_0^{\alpha_0}}{\Gamma(\alpha_0)\beta'^{\alpha'}} \frac{(1/2)^{(n_g - p)/2}}{\Gamma\left((n_g - p)/2\right)} R_g^{(n_g - p)/2 - 1} \\
&= \frac{\Gamma((n_g - p)/2 + \alpha_0)\beta_0^{\alpha_0}}{\Gamma(\alpha_0)\left(\beta_0 + R_g/2\right)^{(n_g - p)/2 + \alpha_0}} \frac{(1/2)^{(n_g - p)/2}}{\Gamma\left((n_g - p)/2\right)} R_g^{(n_g - p)/2 - 1} \\
&= \frac{\Gamma((n_g - p)/2 + \alpha_0)\beta_0^{\alpha_0}}{\Gamma(\alpha_0)} \frac{(1/2)^{(n_g - p)/2}}{\Gamma\left((n_g - p)/2\right)} \frac{R_g^{(n_g - p)/2 - 1}}{\beta_0^{(n_g - p)/2 + \alpha_0}\left(1 + R_g/(2\beta_0)\right)^{(n_g - p)/2 + \alpha_0}} \\
&= \frac{(1/2)^{(n_g - p)/2}}{\mathcal{B}\left((n_g - p)/2, \alpha_0\right)\beta_0^{(n_g - p)/2}} \frac{R_g^{(n_g - p)/2 - 1}}{\left(1 + R_g/(2\beta_0)\right)^{(n_g - p)/2 + \alpha_0}} \qquad (1)
\end{aligned}$$

where $\mathcal{B}$ is the beta function. This is recognized as the kernel of a (scale) F-distribution. Since $1 + R_g$ is raised to the $-((n_g - p)/2 + \alpha_0)$, R_g is raised to the $(n_g - p)/2 - 1$ power, and R_g is divided by $2\beta_0$, we identify the parameters of the scale-F distribution d_1, d_2, σ as

$$\begin{aligned}\frac{d_1 + d_2}{2} &= [(n_g - p)/2 + \alpha_0]\\ \frac{d_1}{2} - 1 &= (n_g - p)/2 - 1\\ \frac{d_1}{d_2\sigma} &= 1/(2\beta_0).\end{aligned}$$

Solving this system gives

$$\begin{aligned}d_1 &= n_g - p\\ d_2 &= 2\alpha_0\\ \sigma &= \frac{\beta_0(n_g - p)}{\alpha_0}.\end{aligned}$$

Working backwards from a $F\left(n_g - p, 2\alpha_0, \frac{\beta_0(n_g-p)}{\alpha_0}\right)$ distribution, we would have that

$$f(R_g) = \frac{1}{\mathcal{B}\left(\frac{(n_g-p)}{2}, \alpha_0\right)} \left(\frac{(n_g - p)}{2\alpha_0}\right)^{(n_g-p)/2} \left[\frac{R_g\alpha_0}{\beta_0(n_g - p)}\right]^{(n_g-p)/2-1} \left[1 + \frac{R_g}{(2\beta_0)}\right]^{-(n_g-p+2\alpha_0)/2} \frac{\alpha_0}{\beta_0(n_g - p)},$$

which after some algebra verifies to be equivalent to equation 1.

2.1.1. *Maximum Likelihood Estimators.* Equation 1 can be used as the basis for maximum likelihood estimation of α_0, β_0. Dropping constants that do not depend on the parameters, the log-likelihood and score functions have the form

$$\begin{aligned}\mathcal{L}(\alpha_0,\beta_0) &= -\log\mathcal{B}\left((n_g - p)/2, \alpha_0\right) - \frac{n_g - p}{2}\log\beta_0 - \log\left(1 + R_g/(2\beta_0)\right)\left((n_g - p)/2 + \alpha_0\right)\\ \mathcal{L}_{\alpha_0} &= \psi\left((n_g - p)/2 + \alpha_0\right) - \psi\left(\alpha_0\right) - \log\left(1 + R_g/(2\beta_0)\right)\\ \mathcal{L}_{\beta_0} &= \frac{\alpha_0 R_g - (n_g - p)\beta_0}{R_g\beta_0 + 2\beta_0^2},\end{aligned}$$

where ψ is the digamma function $\frac{d\Gamma(x)}{dx}$. This likelihood may be maximized numerically, eg, using the *optim* function in R.

2.1.2. *Posterior MLE for τ_g.* Given estimates α_0, β_0 derived by MLE, then the posterior distribution of τ_g is Gamma-rate with parameters $\alpha' = \alpha_0 + (n_g - p)/2$ and $\beta' = \beta_0 + R_g/2$. The log-likelihood and score for τ_g is

$$\begin{aligned}\mathcal{L}(\tau_g) &= (\alpha' - 1)\log\tau_g - \tau_g\beta'\\ \mathcal{L}_{\tau_g} &= \frac{\alpha' - 1}{\tau_g} - \beta'\end{aligned}$$

which implies that

$$\hat{\tau_g} = \frac{\alpha' - 1}{\beta'}$$

which has an interpretation in terms of pseudo-observations as follows

$$\begin{aligned} 1/\hat{\tau_g} &= \frac{R_g/2 + \beta_0}{\alpha_0 + (n_g - p)/2} = \frac{R_g}{n_g - p}\frac{n_g - p}{2\alpha_0 + n_g - p} + \frac{\beta_0}{\alpha_0}\frac{2\alpha_0}{2\alpha_0 + n_g - p} \\ &= \left(\tau_g^{-1}\right)^{\text{MLE}} \lambda + 1/\tau_0(1-\lambda) \end{aligned}$$

noting that $\left(\tau_g^{-1}\right)^{\text{MLE}} = \frac{R_g}{n_g - p}$ would be the typical MLE of the variance τ_g^{-1} and that $\tau_0 = \alpha_0/\beta_0$ would be the MLE of τ using only the prior information. This final formulation of the shrunken precision as a convex combination of the MLE and the global value τ_0 is used in practice.

2.2. **Bayesian logistic regression for discrete component.** In logistic regression, when the binary outcome can be perfectly predicted by a covariate (or linear combination of covariates), then "linear separation" is said to be present, and parameter estimates will diverge towards $\pm\infty$ while the Fisher information becomes singular. (In contrast, if even a single cell were to violate this linear separation, then the Fisher Information would be invertible.) Yet cases with linear separation are of particular interest, since a gene that so sharply changes by condition is noteworthy. To accommodate this scenario, we apply a Bayesian logistic regression procedure available in the `bayesglm` function in the `R` package `arm`. A Cauchy distribution prior centered at zero for the regression coefficients results in maximum aposteriori (MAP) estimates nearly identical to the maximum likelihood estimates when linear separation is not present. Under linear separation the Bayesian MAP estimate is finite, with non-singular Hessian about the MAP (providing an estimate of the statistical precision, akin to the Fisher information.) Favorable small-sample frequentist properties have also been described in Gelman et al. (2008).

3. Residual Analysis

3.1. **Deviance Residuals.** The hurdle model, in general, provides two residuals–one for the discrete component and one for the continuous. Standardized deviance residuals are calculated for the discrete and continuous component separately, then we combine the residuals by averaging them. If a cell is unexpressed, then its residual is missing and it is omitted from the average.

For a given gene, and model component (discrete or continuous) the residual deviance D is -2 times the maximized log-likelihood, (centered so that $D = 0$ when every observation has its own mean parameter). The deviance can be written as a sum of -2 times the log-likelihood of each observation, or

$$D = \sum_{i=1}^{N} d_i.$$

The deviance residual is defined as

$$r_i = \text{sign}(y_i - \hat{y_i})\sqrt{d_i}$$

and the standardized deviance residual given by $r_d' = \frac{r_d}{\sqrt{1-h_i}}$ where h_i is the leverage associated with observation i.

The combined deviance residual for cell i is the average of the standardized discrete and continuous deviance residuals for the cell if both are present, otherwise it is only the standardized discrete residual.

4. Gene Set Enrichment

Fix a gene module, ie, a collection of gene indices. Let $g = 1, \ldots, G_0, \ldots, G$ index the G genes measured, with $G - G_0$ genes in the *test set* (set of interest) and G_0 genes in the *null set* (outside the set of interest). We assume that the following hurdle linear models

$$E\left(\mathbf{Y_g} | \mathbf{Y_g} > 0\right) = x\beta_g + Z\eta_g$$
$$\text{logit } E(\mathbf{Y_g} > 0) = x\beta'_g + Z\eta'_g$$

have been fit for $g = 1, \ldots, G$. Here x is a simple covariate of interest (scalar in each observation) while Z is all other covariates (potentially a vector in each observation). The competitive gene set enrichment test considers the average coefficient of interest in the test and null sets:

$$\hat{\theta} = \frac{1}{G - G_0} \sum_{g=G_0+1}^{G} \hat{\beta}_g$$

$$\hat{\theta}' = \frac{1}{G - G_0} \sum_{g=G_0+1}^{G} \hat{\beta}'_g$$

$$\hat{\theta}_0 = \frac{1}{G_0} \sum_{g=1}^{G_0} \hat{\beta}_g$$

$$\hat{\theta}'_0 = \frac{1}{G_0} \sum_{g=1}^{G_0} \hat{\beta}'_g$$

and forms the test statistics

$$Z = \frac{\hat{\theta} - \hat{\theta}_0}{\sqrt{\hat{\text{Var}}(\hat{\theta}) + \hat{\text{Var}}(\hat{\theta}_0)}}$$

with Z' formed analogously. The goal is to form an estimate of $\hat{\text{Var}}(\hat{\theta})$ and $\hat{\text{Var}}(\hat{\theta}_0)$. Since, for example,

$$\begin{aligned}\hat{\text{Var}}(\hat{\theta}_0) &= \text{Var}\left[\frac{1}{G_0}\sum_{g=1}^{G_0}\hat{\beta}_g\right] \\ &= \frac{1}{G_0^2}\left[\sum_{g=1}^{G_0}\text{Var}\hat{\beta}_g + 2\sum_{1\le g<h\le G_0}\text{Cov}\left(\hat{\beta}_g, \hat{\beta}_h\right)\right]\end{aligned}$$

it suffices to find some estimate of the genewise covariance matrix $\hat{\Sigma} \in \mathbb{R}^{G\times G}$ for $\hat{\beta}$. We chose to accomplish this by bootstrap. Repeat R times: sample cells with replacement and generate an expression matrix Y^*, and refit the hurdle linear model providing coefficients $\hat{\beta}^*$ (and $\hat{\beta}'^*$). Collect the the bootstrapped coefficients in matrix $\hat{\boldsymbol{\beta}}^* \in \mathbb{R}^{R\times G}$. Estimate $\hat{\Sigma}$ via the sample covariance on $\hat{\boldsymbol{\beta}}^*$.

4.1. **Implementation Notes.** We find that the bootstrapped covariances converge rather quickly, and $R = 100$ typically more than suffices. An adjustment to Z to account for Monte Carlo variation in the bootstrap estimate $\hat{\Sigma}$ is also available by comparing Z to a t-distribution with degrees of freedom determined through Welch's approximation on R effective observations.

Note that the full covariance matrix estimate $\hat{\Sigma}$ never need be explicitly formed (since it is potentially memory intensive). Rather we accumulate the sum over the $(G - G_0)(G - G_0 + 1)/2$ inner products on the genes in the test set, to yield $\hat{\text{Var}}(\theta)$. A working estimate of $\hat{\text{Var}}(\theta_0)$ is updated by adding and subtracting only the covariances that have changed as G_0 changes between modules.

4.2. **Combining Z and Z'.** Stouffer's method for combining Z-scores is used to form the composite $\bar{Z} = (Z + Z')/\sqrt{2}$.

5. Simulation

In order to assess the effect of the including/excluding CDR effects when modeling single-cell gene expression data, we simulated $\log_2$TPM expression matrix with 2500 genes where 100 genes are differentially expressed for sample size of 100 in each of two stimulation conditions. We tested four scenarios: one with no CDR effect in the simulated data generating process; and three others with varying levels of confounding between CDR and stimulation effect. The four scenarios are described in Table 1 and depicted in Figure 4. The parameters in the data generating model were chosen to mimic the the observed features of the MAIT experiment, as described below.

The results based on 100 replication is summarized by the ROC curves in Figure 4 showing the importance of controlling for CDR when there is a CDR effect in the data generating process. This is especially important when there is confounding between the stimulation and the CDR, as ignoring the CDR effect would typically inflate the type I error rate. At the same time our results also indicates the robustness of our proposed

model for including CDR even when there is no CDR effect in the data generating process, promoting the inclusion of CDR as a default model.

5.1. **Data generating protocol.** We set the sample size $N = 200$, the number of genes J=2500 and defined the stimulation indicator s as 0 for first 100 cells and 1 for the last 100. Given stimulation indicator s we generated the data accordingly:

$$\begin{aligned}
\tau_j^2 &\sim Gamma(a_0, b_0) \\
\mathrm{CDR}_i &\sim (1 - s_i)\mathrm{Beta}(a_u, b_u) + s_i\mathrm{Beta}(a_s, b_s) \\
z_{ij}|\mathrm{CDR}_i &\sim \mathrm{Bernoulli}(\mathrm{logit}^{-1}(\mu_j^d + \alpha_j^d s_i + \beta_j^d \mathrm{CDR}_i)) \\
y_{ij}|z_{ij}, \mathrm{CDR}_i &\sim z_{ij}\mathrm{N}(\mu_j^c + \alpha_j^c s_i + \beta_j^c \mathrm{CDR}_i, 1/\tau_j^2)
\end{aligned}$$

The coefficients μ, α, and β were generated from a Normal distribution with hyper parameters based on the distribution of these quantities observed in the MAIT experiment. We also set $\alpha_j = 0$ for $j > 100$, since only the first 100 genes are differentially expressed (i.e. have a non-zero treatment effect). The precision hyperparameters a_0 and b_0 are set to the point estimates found in the MAIT experiment. The code with all the simulation details can be found in `AdditionalAnalyses.Rmd`.

Table 1. Hyper parameter settings for CDR generation model.

	strong confounding	moderate confounding	no confounding
a_u	4	6	8
b_s	16	14	12
a_s	12	10	8
b_s	8	10	12

6. R packages MAST and MASTDataPackage

6.1. **R data package MASTDataPackage.** We compiled the data used in this paper into an R data package, containing all data as R objects to version control the dataset for convenient reproducibility. The data package can be installed using the devtools package, as follows,

```
devtools::install_github("RGLab/MASTdata")
```

After installation, the package can be loaded as a standard R package, and the data available as `SingleCellAssay` object.

```
library(MASTDataPackage)
data(MASTDataPackage)
```

Analysis described in the main paper and the supplementary figure are available as the vignette of `MASTDataPackage`. The MAIT cells and mouse dendritic cells analysis are accessible by

```
vignette("MAIT analysis)
```

and

```
vignette("mDC analysis)
```

respectively on the R terminal.

References

Blankenberg, D., A. Gordon, G. Von Kuster, N. Coraor, J. Taylor, A. Nekrutenko, et al. (2010). Manipulation of fastq data with galaxy. *Bioinformatics 26*(14), 1783–1785.

De Wit, H., D. Hoogstraten, R. Halie, and E. Vellenga (1996). Interferon-gamma modulates the lipopolysaccharide-induced expression of ap-1 and nf-kappa b at the mrna and protein level in human monocytes. *Experimental hematology 24*(2), 228–235.

Gelman, A., A. Jakulin, M. G. Pittau, and Y.-S. Su (2008). A weakly informative default prior distribution for logistic and other regression models. *The Annals of Applied Statistics*, 1360–1383.

Gentleman, R. C., V. J. Carey, D. M. Bates, B. Bolstad, M. Dettling, S. Dudoit, B. Ellis, L. Gautier, Y. Ge, J. Gentry, et al. (2004). Bioconductor: open software development for computational biology and bioinformatics. *Genome biology 5*(10), R80.

Kharchenko, P. V., L. Silberstein, and D. T. Scadden (2014). Bayesian approach to single-cell differential expression analysis. *Nature methods 11*(7), 740–742.

Li, B. and C. N. Dewey (2011). Rsem: accurate transcript quantification from rna-seq data with or without a reference genome. *BMC bioinformatics 12*(1), 323.

Padovan-Merhar, O., G. P. Nair, A. G. Biaesch, A. Mayer, S. Scarfone, S. W. Foley, A. R. Wu, L. S. Churchman, A. Singh, and A. Raj (2015). Single mammalian cells compensate for differences in cellular volume and dna copy number through independent global transcriptional mechanisms. *Molecular cell 58*(2), 339–352.

Shalek, A. K., R. Satija, J. Shuga, J. J. Trombetta, D. Gennert, D. Lu, P. Chen, R. S. Gertner, J. T. Gaublomme, N. Yosef, et al. (2014). Single-cell rna-seq reveals dynamic paracrine control of cellular variation. *Nature*.

7. Supplementary Figures

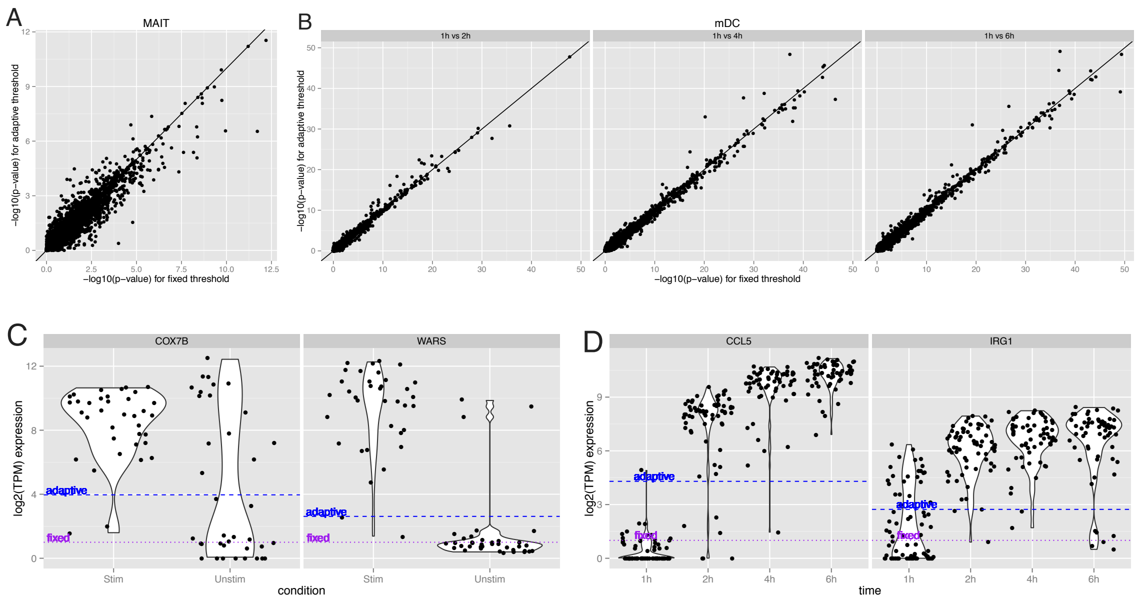

Figure 1. Scatter plot of p-values for differential expression from adaptive and fixed thresholding on the A) MAIT and B) mDC data sets, demonstrating robustness to the thresholding method. Two selected genes from each data set, with large differences in p-values between fixed and adaptive thresholding in C) MAIT and D) mDC, are genes that exhibit substantial bimodality and our adaptive thresholding appears preferable.

Table 2. Standard deviations of module scores for stimulated and non-stimulated MAIT cells

set	Unstim	Stim
signaling in T cells (II) (M35.1)	1.2301815	2.1008091
chaperonin mediated protein folding (I) (M204.0)	0.6746352	1.4881732
respiratory electron transport chain (mitochondrion) (M238)	0.9977339	1.0474434
AP-1 transcription factor network (M20)	1.5442480	1.0009314
proteasome (M226)	1.0019731	1.8099783
cell cycle and growth arrest (M31)	1.4221590	0.7827468
chaperonin mediated protein folding (II) (M204.1)	0.8101438	1.6062428
purine nucleotide biosynthesis (M212)	0.7082552	1.5784755
spliceosome (M250)	1.1026929	1.3047972

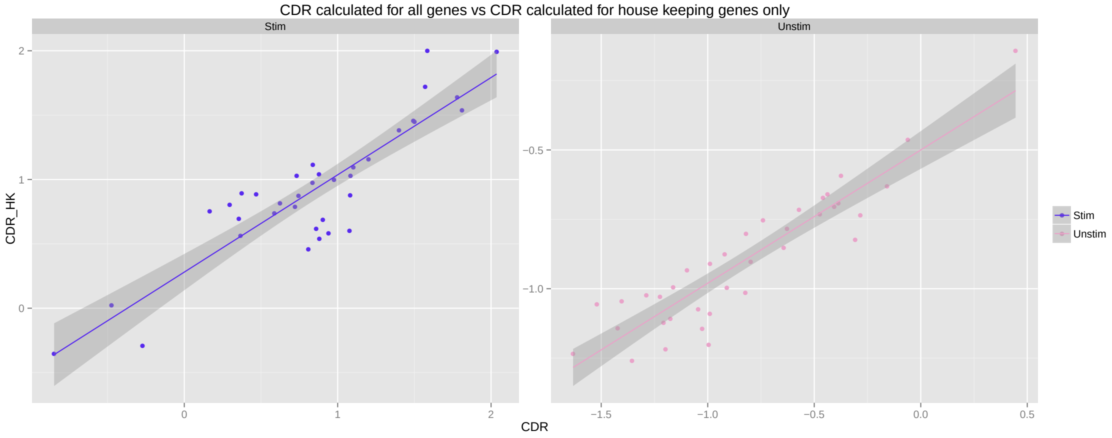

Figure 2. Scatter plot of normalized (scaled to unit variance and zero mean) CDR (cellular detection rate) calculated from all genes vs. the CDR calculated from housekeeping genes (Padovan-Merhar et al., 2015), for stimulated A) and unstimulated B) MAIT cells. The estimated CDRs are linearly related within each condition.

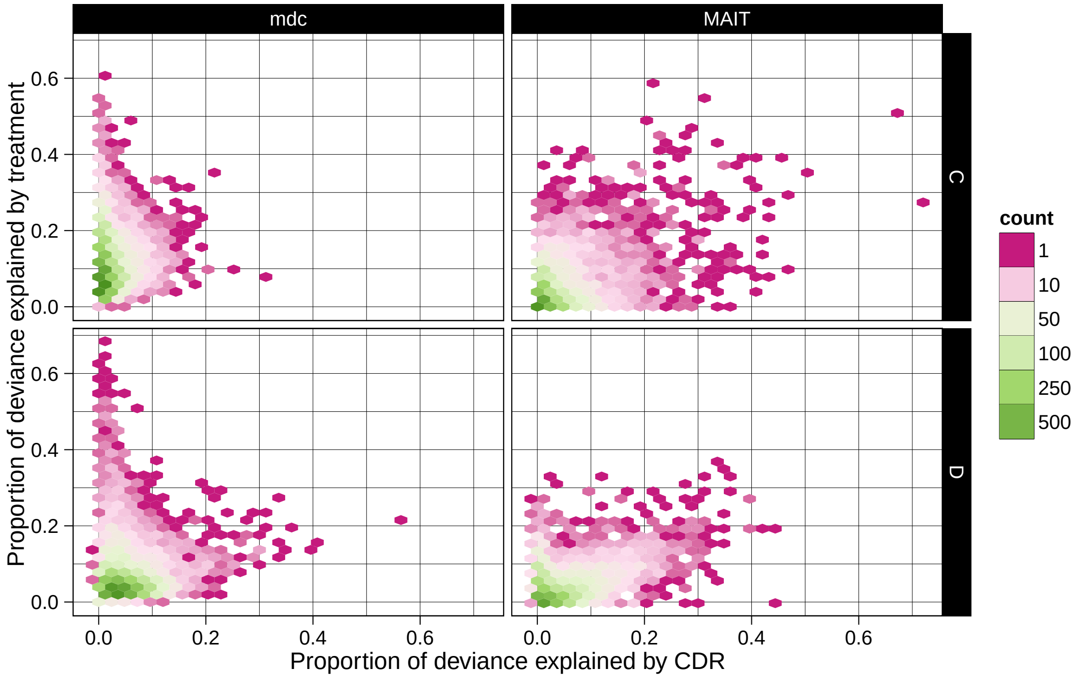

FIGURE 3. Amount of variability, measured as percent of null model deviance, attributed to the CDR effect vs. the treatment effect, in each dataset. The CDR accounts for 5.2% of the variability in the MAIT and 4.8% of the variability in the mDC data sets for the average gene. Greater than 9% of the variability is attributed to over 10% of genes in both data sets. CDR contributes the most variability to the discrete component in both data sets and more so in the MAIT data than the mDC data.

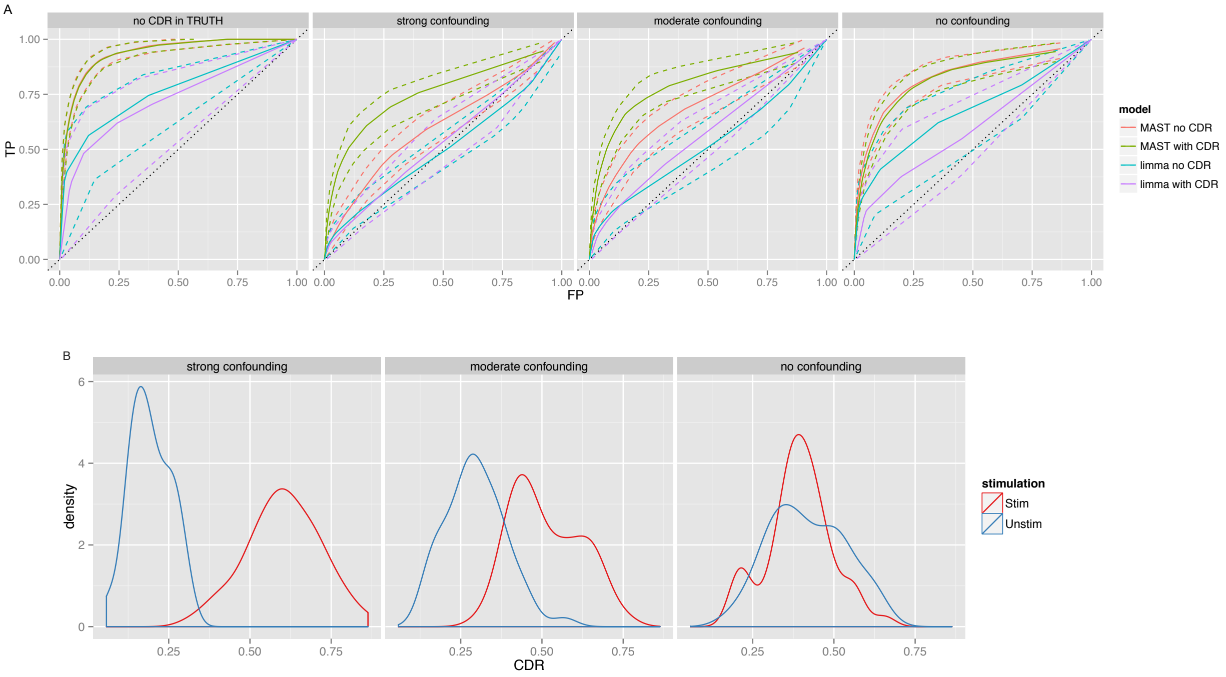

Figure 4. Effect of CDR and confounding with treatment using different methods. A) ROC curve comparing the effect of controlling for CDR in the MAST model. The solid line is the median and the top and the bottom dashed line represents the 95 and 5 percent quantile. The result indicates that inclusion of CDR improves the performance when there is confounding between the CDR and stimulation and performs nearly the same when there is no confounding or when there is no CDR effect in the data generating model. B) Density plot of generated CDR values across cells using the three levels of confounding between the stimulation and the CDR effects.

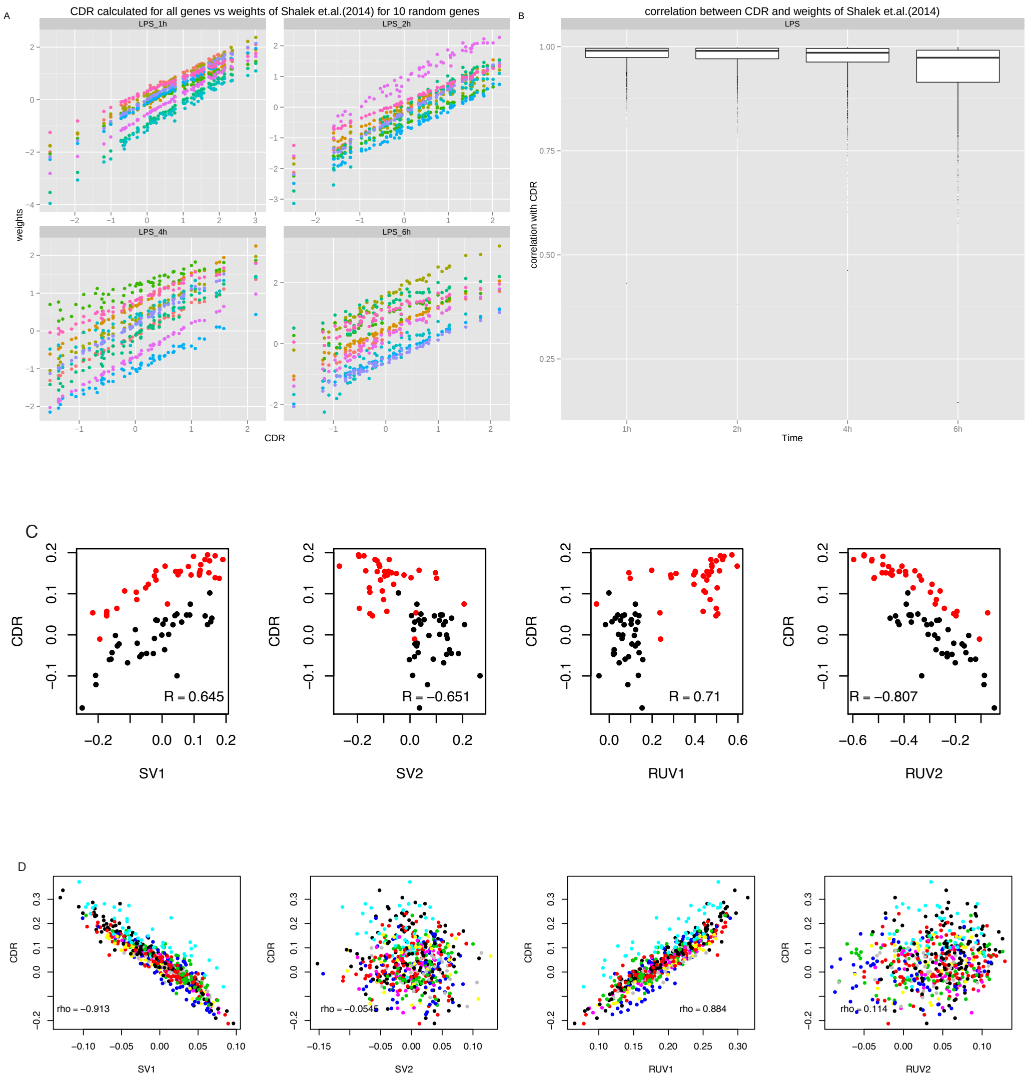

FIGURE 5. Comparison of the empirical CDR (centered and scaled) and other correction methods, the cell by gene weights of Shalek et. al., and RUV and SVA. The CDR and Shalek et. al. weights are correlated, in fact generally just shifted by a constant (panel A, in a random subsample of genes, each in a different color), and the correlation coefficient is nearly unity (panel B). The location shift between the CDR and Shalek et. al. weights would be absorbed by the intercept term in the logistic regression. C) Scatterplots of CDR vs. the first and second SVA and RUV components. Treatment groups are shown in different colors. The first SVA and second RUV components are associated with CDR. D) In the mDC data, the first SVA and RUV components are correlated with CDR.

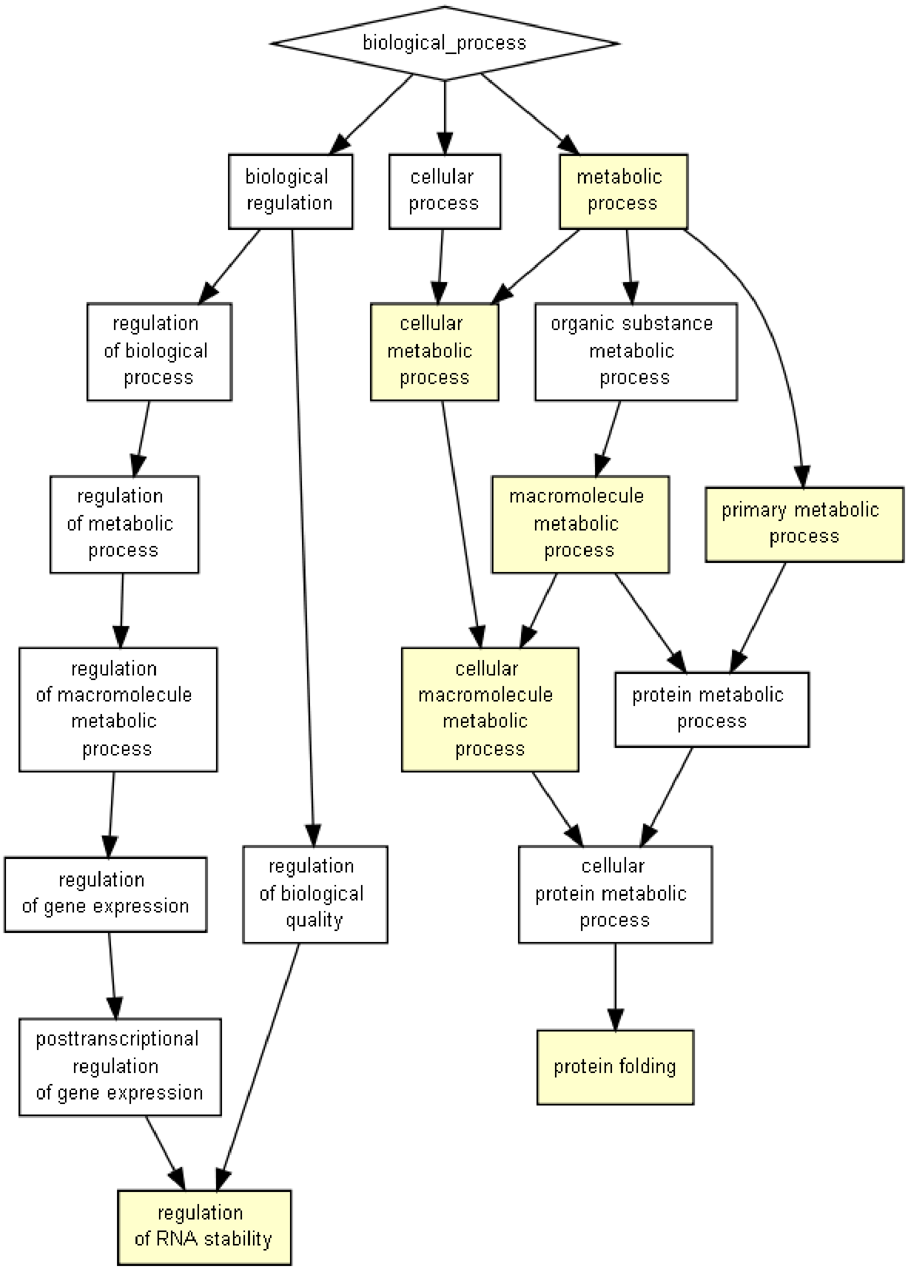

Figure 6. Gene Ontology Enrichment Analysis using the GOrilla online tools for the set of genes not detected as differentially expressed in the MAIT data set when the CDR is included in the MAST linear model.

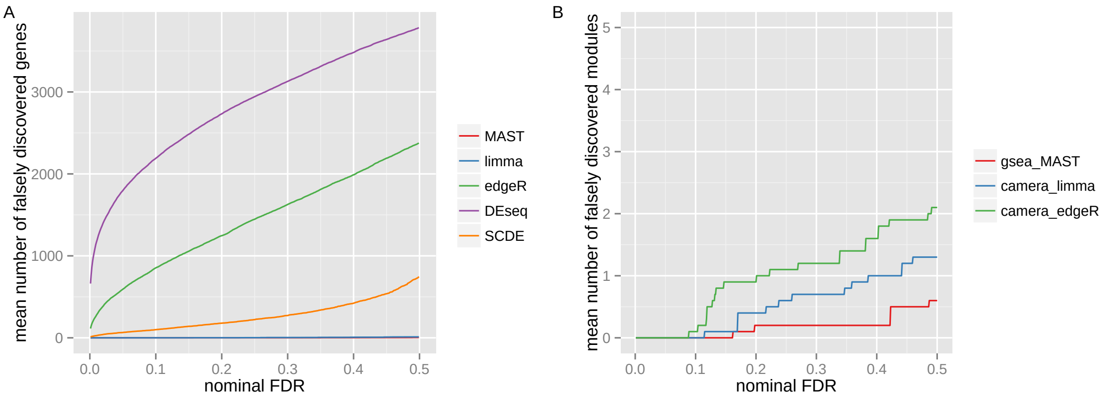

FIGURE 7. False discoveries in genes (A) and modules (B) based on numeric permutation experiments for various methods. The unstimulated MAIT cells were permuted into two subsets, and were tested for differential expression under the Hurdle model (MAST), Limma, edgeR, and DEseq. In this scenario, any gene discovered is an *a priori* false discovery, so the number of false discoveries is plotted against the FDR-adjusted significance. We show the average values from ten permutations.

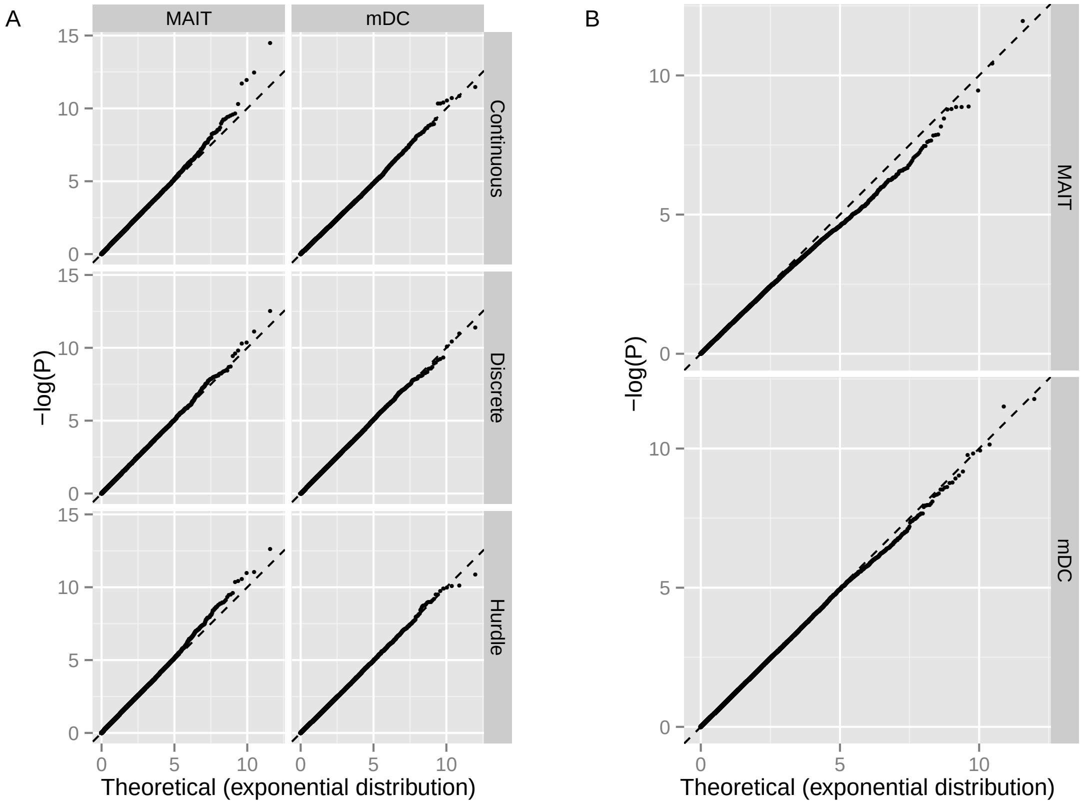

FIGURE 8. The distribution of log p-values in permuted datasets is compared to its expected Exponential(1) distribution in (A) the hurdle model and (B) Normal-theory t-tests on the same data. In the smaller MAIT dataset ($N = 73$) the Hurdle is inflated in the tail of the test statistic, producing an additional .6 rejections per 1,000 tests at $\alpha = 10^{-3}$. The t-test is deflated, yielding .5 too few rejections per 1,000 tests at $\alpha = 10^{-3}$

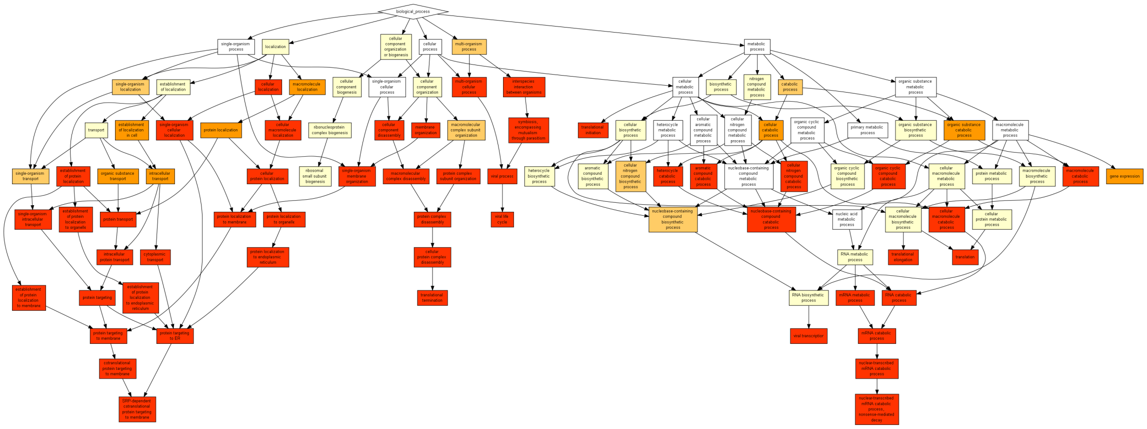

Figure 9. Gene Ontology Enrichment Analysis using the GOrilla online tools for the set of genes detected as differentially expressed by DESeq but not by MAST.

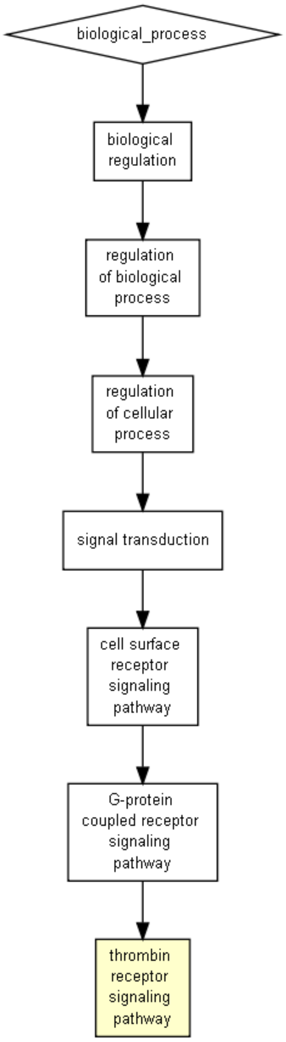

FIGURE 10. Gene Ontology Enrichment Analysis using the GOrilla online tools for the set of genes detected as differentially expressed by edgeR but not by MAST.

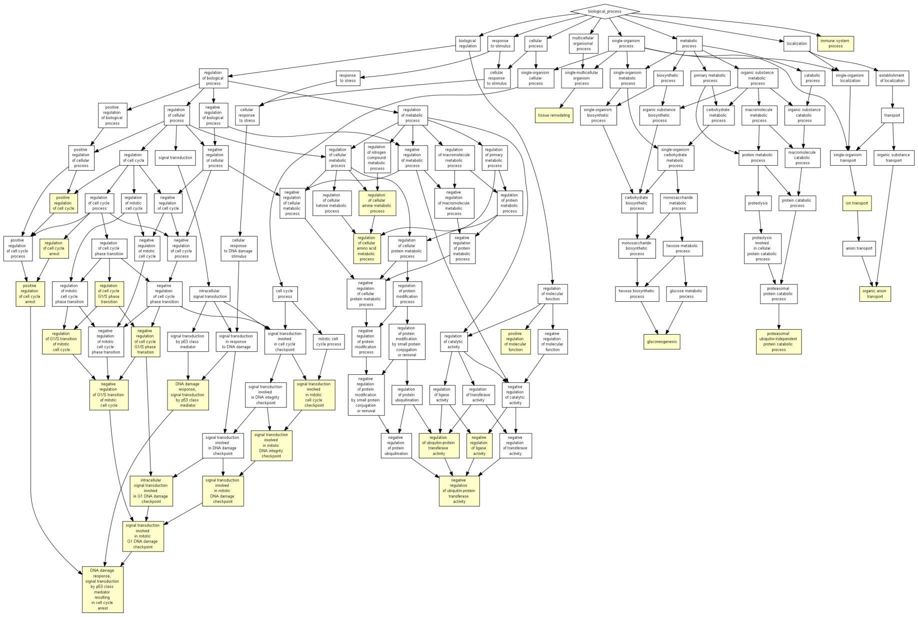

FIGURE 11. Gene Ontology Enrichment Analysis using the GOrilla online tools for the set of genes detected as differentially expressed by Limma but not by MAST.

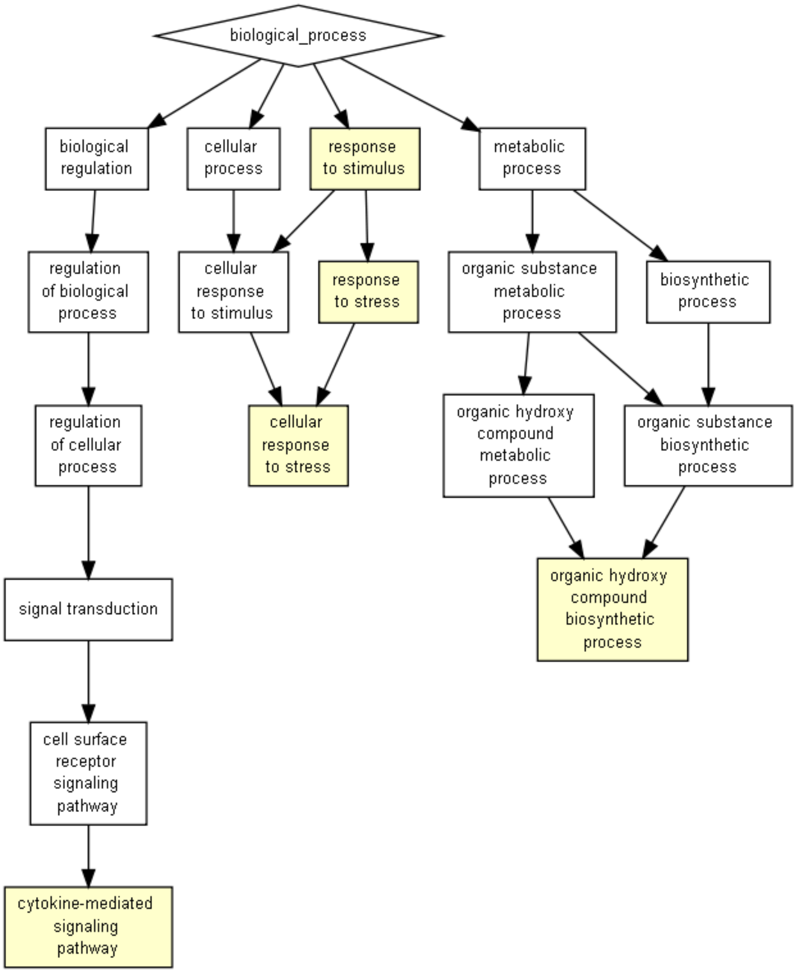

Figure 12. Gene Ontology Enrichment Analysis using the GOrilla online tools for the set of genes detected as differentially expressed by SCDE but not by MAST.

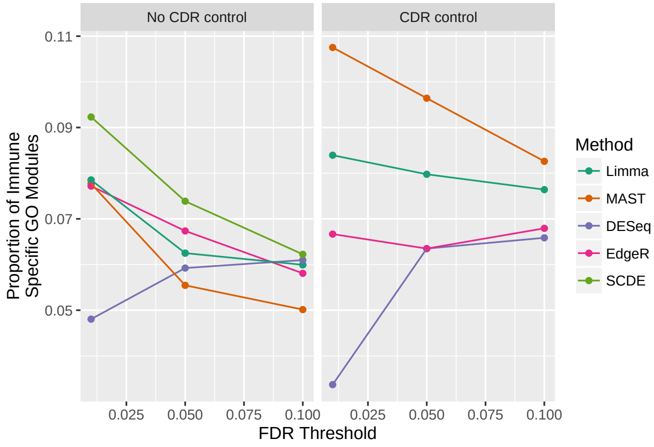

FIGURE 13. Proportion of immune-specific GO modules amongst all GO modules enriched in differentially expressed genes in the MAIT data set. Immune-specific GO modules were defined to be terms with experimental evidence codes within the Biological Process ontology that were descendants in the GO graph of the Immune System Process term. Differential expression of genes was determined at three increasing false discovery rate thresholds, and then GO enrichment in differentially expressed genes was tested using the hypergeometric distribution, calling significant enrichment at the 1% FDR level. Inclusion of the CDR in the model for differential expression increases the rate of detection of immune specific modules for the MAST and Limma methods. Among models that do not adjust for CDR, SCDE has highest specificity, but is dominated by MAST under CDR adjustment (SCDE cannot adjust for covariates, so was omitted from the CDR models).

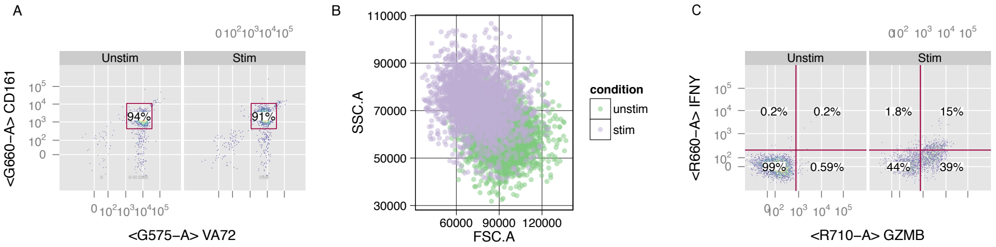

FIGURE 14. Post-sort experiments via flow cytometry show that the sorted cell populations were over 90% pure MAITs (Figure A), and exhibited a change in cell size upon stimulation (Figure B) and that up to 44% of stimulated MAITs did not respond to cytokine stimulation (Figure C).

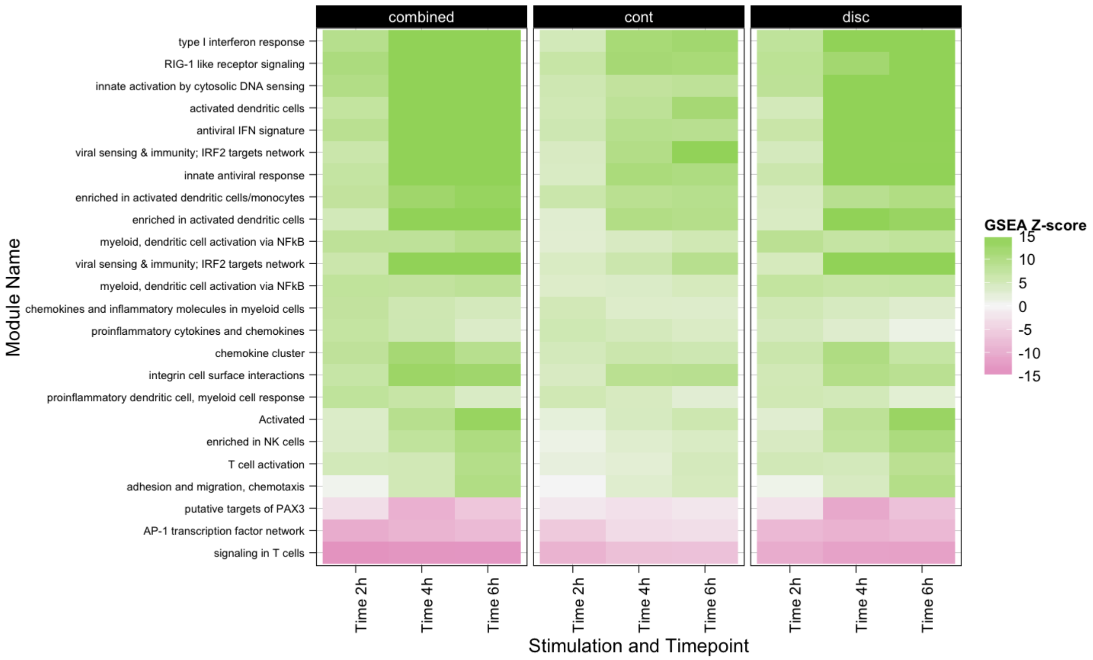

Figure 15. Gene set enrichment analysis of the mDC data set, LPS stimulated cells using the BTM (blood transcriptional modules) of Li et. al. Decreased expression for AP-1 transcriptional network genes is observed after LPS stimulation, consistent with previous findings in the literature (De Wit et al., 1996). Type-1 interferon response and antiviral IFN modules are among the most significantly enriched and are consistent with the findings of the original publication (Shalek et al., 2014) .

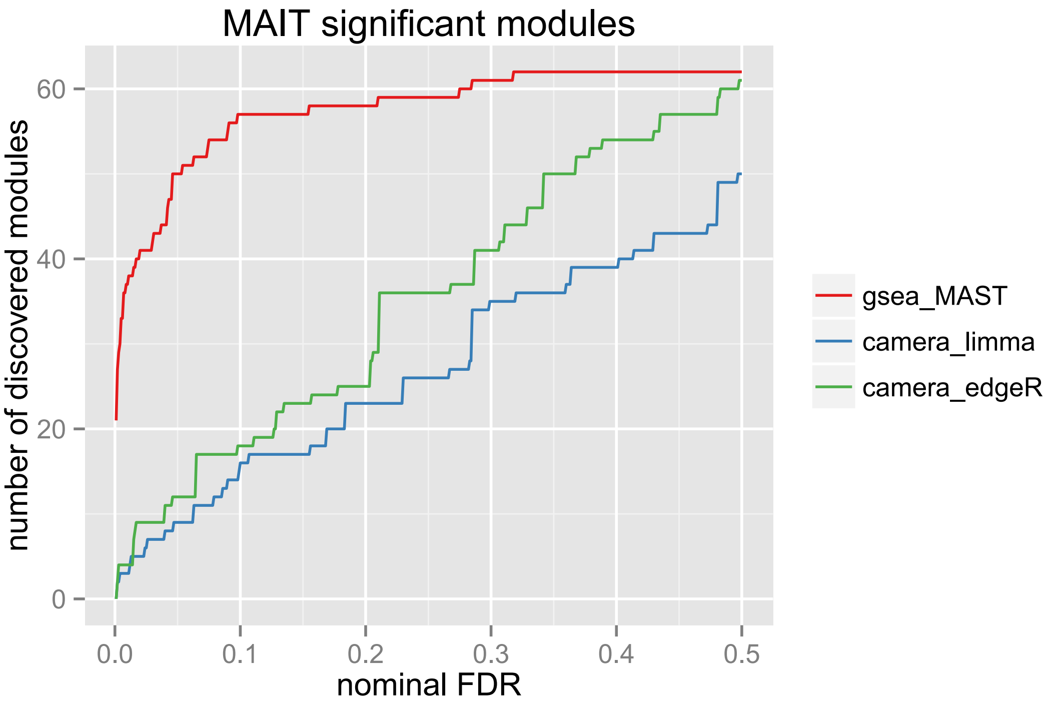

FIGURE 16. Number of modules discovered plotted against FDR-adjusted significance of the module. MAST-based GSEA detects more modules than other methods.

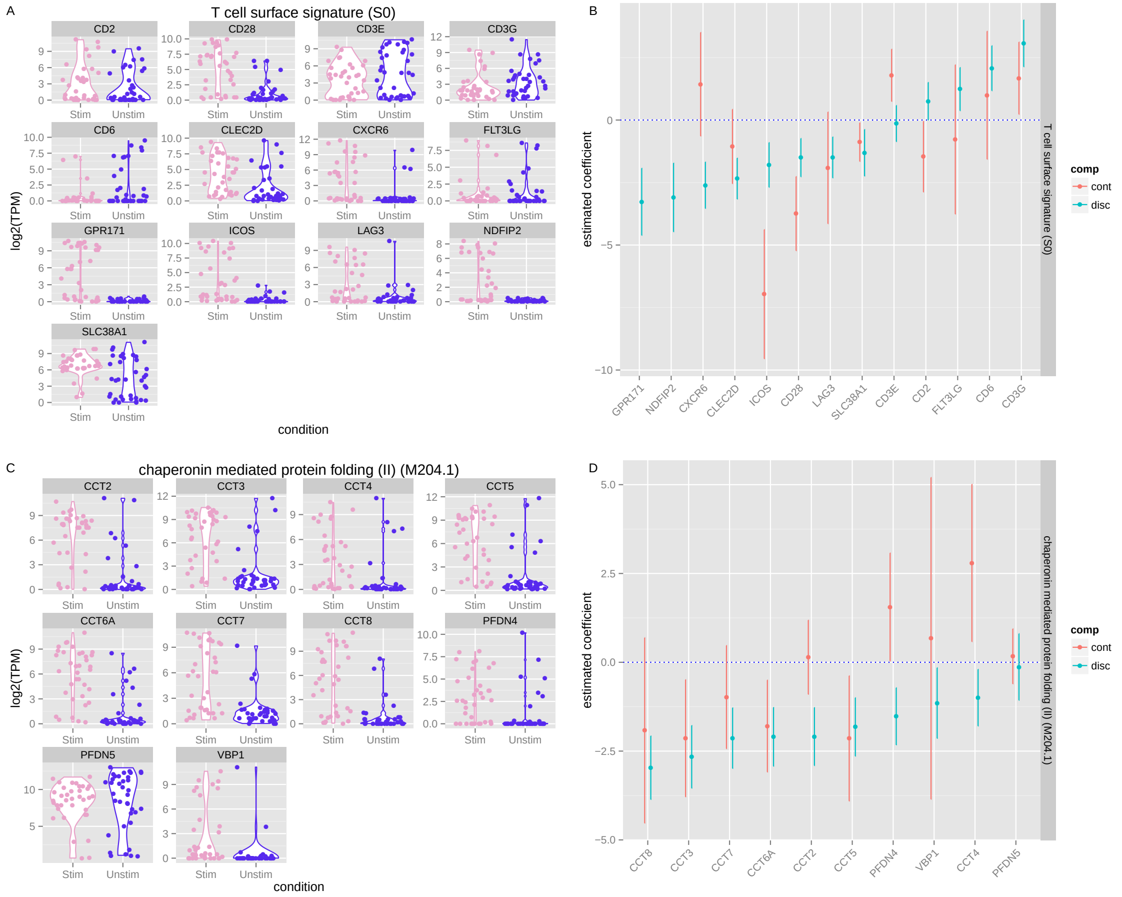

FIGURE 17. Comparison of raw expression values ($\log_2$TPM) and coefficients estimates (Unstimulated as reference) of modules identified as differentially expressed using MAST GSEA but not with CAMERA. Differences in the expression profile are evident, however CAMERA failed to detect them. A) Violin plots showing the expression of genes in the "T-cell surface signature" module. B) Model coefficient estimates for the genes in the "T-cell surface signature" module from GSEA, with 95% confidence intervals, from the discrete and continuous components of the model. C) Violin plots showing the expression of genes in the "chaperonin mediate protein folding" module. D) Model coefficient estimates for the genes in the "chaperonin mediate protein folding" module from GSEA, with 95% confidence intervals, from the discrete and continuous components of the model.

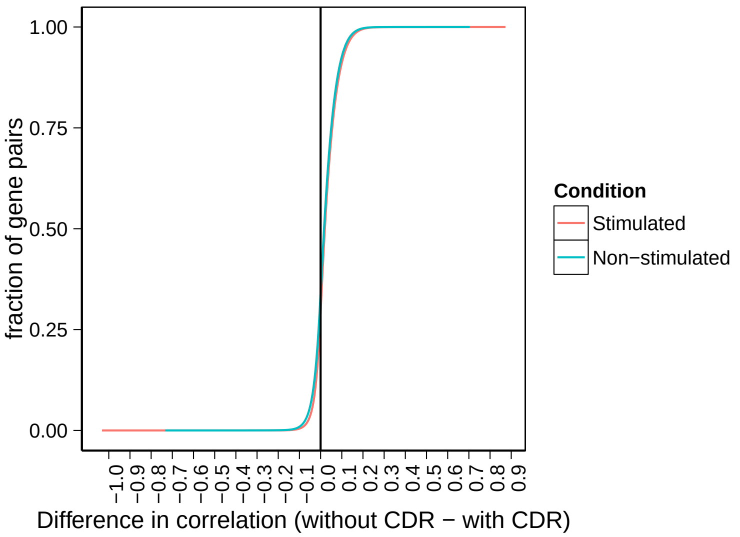

FIGURE 18. Distribution of *changes* in pairwise correlations of MAST model residuals after adjusting for CDR. Controlling for the CDR effect modestly reduces the background correlation in the median gene in both stimulated cells (red) and unstimulated (blue). Each distribution consists of approximately 1.8 million gene pairs.

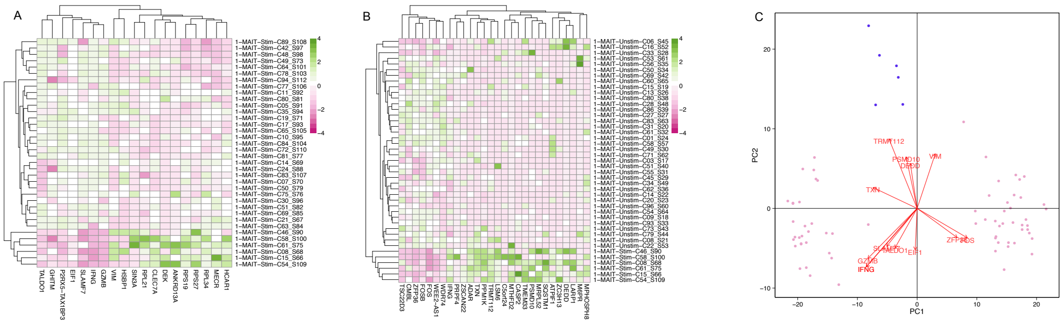

FIGURE 19. The six stimulated MAIT cells that did not exhibit an expression profile indicative of activation are shown in comparison to A) other stimulated MAITs and B) unstimulated MAITs. Differentially expressed genes between these six cells and the stimulated but activated and non-stimulated cells are shown, identified using MAST at a q-value of 15% and fold change threshold of at least 2. Panel C) shows PCA of the MAITs based on the differentially expressed genes. 13 selected gene with largest loadings discriminating between the three classes of cells are shown.

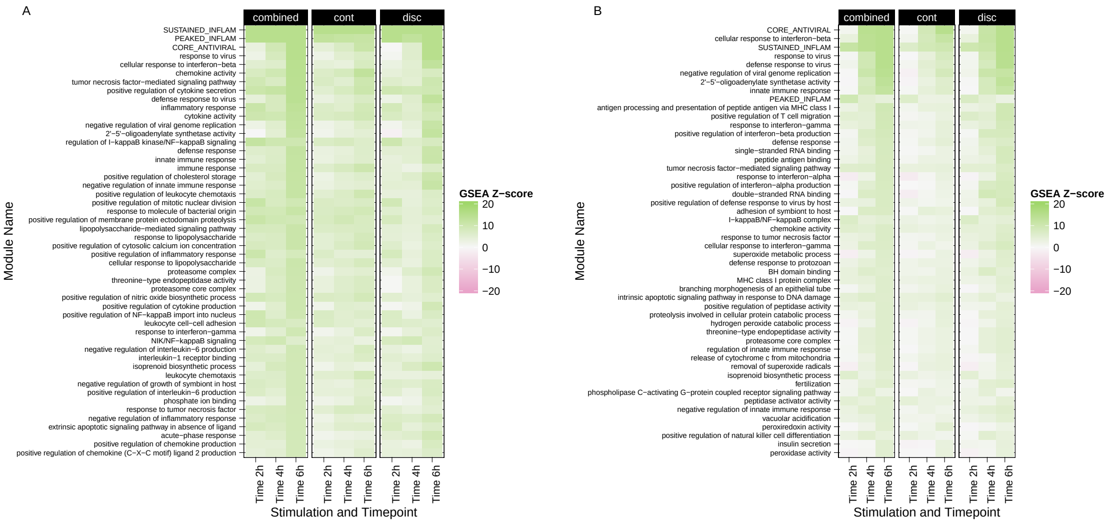

FIGURE 20. Hurdle model GSEA of the PAM (A) and PIC (B) stimulated mDC cells.

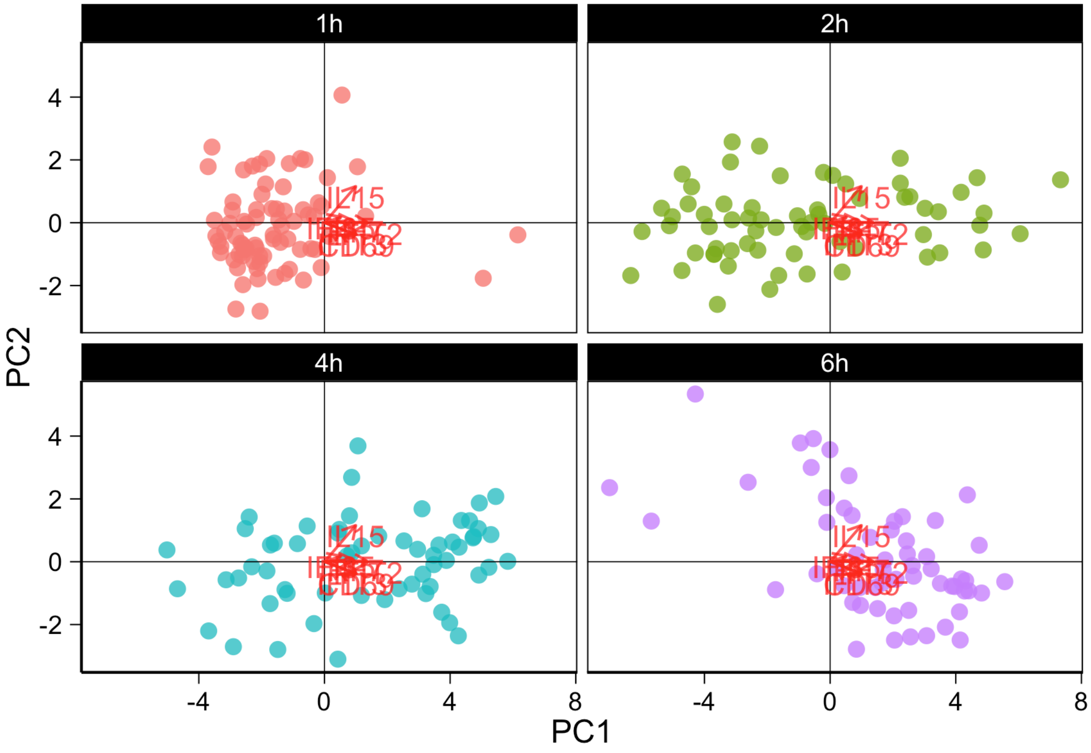

Figure 21. PCA of the model residuals of LPS stimulated cells using the genes in the core antiviral module identified in Shalek et al. (2014) The two "outlier" cells evident at the 1h timepoint correspond to the "early marcher" precocious cells described previously. These results show that these cells exhibit coordinated co-expression of genes in the core antiviral signature at the single-cell level.

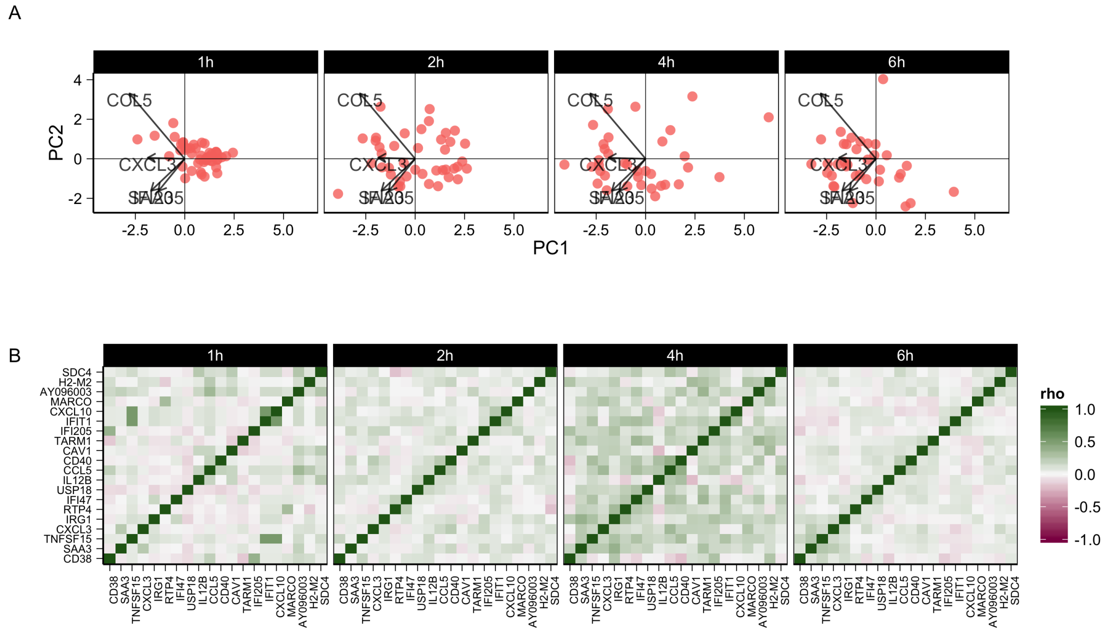

FIGURE 22. Co-expression plot for PAM (synthetic mimic of bacterial lipopeptides) stimulated cells of cells in the mDC data. Panel A in each figure shows principal component analysis (PCA) of the model residuals using the top 100 differentially expressed genes. Cells are faceted by time, which is correlated with the first principal component. Panel B shows heatmaps of the pairwise correlations between genes in the model residuals across cells at each timepoint. The order of genes in the heatmaps is based on clustering at the 6h timepoint.

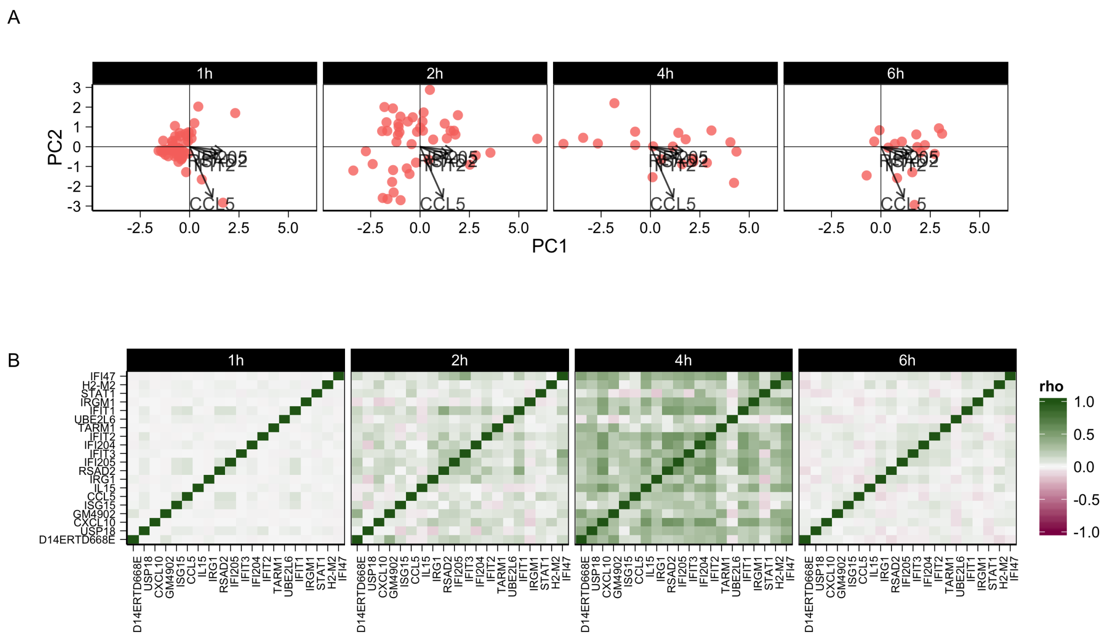

FIGURE 23. Co-expression plot for PIC (viral-like double-stranded RNA) stimulated cells of cells in the mDC data. Panel A in each figure shows principal component analysis (PCA) of the model residuals using the top 100 differentially expressed genes. Cells are faceted by time, which is correlated with the first principal component. Panel B shows heatmaps of the pairwise correlations between genes in the model residuals across cells at each timepoint. The order of genes in the heatmaps is based on clustering at the 6h timepoint.
